# Sensitive and specific detection of Crimean-Congo Hemorrhagic Fever Virus (CCHFV)—Specific IgM and IgG antibodies in human sera using recombinant CCHFV nucleoprotein as antigen in μ-capture and IgG immune complex (IC) ELISA tests

**DOI:** 10.1371/journal.pntd.0006366

**Published:** 2018-03-26

**Authors:** Petra Emmerich, Angela Mika, Ronald von Possel, Anne Rackow, Yang Liu, Herbert Schmitz, Stephan Günther, Kurtesh Sherifi, Barie Halili, Xhevat Jakupi, Lindita Berisha, Salih Ahmeti, Christina Deschermeier

**Affiliations:** 1 Department for Virology, Bernhard Nocht Institute for Tropical Medicine, Hamburg, Germany; 2 Department of Tropical Medicine and Infectious Diseases, Center of Internal Medicine II, University of Rostock, Rostock, Germany; 3 Diagnostics Development Laboratory, Bernhard Nocht Institute for Tropical Medicine, Hamburg, Germany; 4 Faculty of Agricultural and Veterinary Medicine, University of Pristhina “Hasan Prishtina”, Pristhina, Kosovo; 5 University Clinical Center of Kosovo, Infectious Diseases Clinic, Pristhina, Kosovo; 6 Department of Microbiology, National Institute for Public Health of Kosova, Prishtina, Kosovo; 7 University of Prishtina “Hasan Prishtina”, Medical Faculty & University Clinical Center of Kosovo, Infectious Diseases Clinic, Prishtina, Kosovo; International Atomic Energy Agency, AUSTRIA

## Abstract

As the most widespread tick-borne arbovirus causing infections in numerous countries in Asia, Africa and Europe, Crimean-Congo Hemorrhagic Fever Virus (CCHFV, family *Nairoviridae*) was included in the WHO priority list of emerging pathogens needing urgent Research & Development attention. To ensure preparedness for potential future outbreak scenarios, reliable diagnostic tools for identification of acute cases as well as for performance of seroprevalence studies are necessary. Here, the CCHFV ortholog of the major bunyavirus antigen, the nucleoprotein (NP), was recombinantly expressed in *E*.*coli*, purified and directly labeled with horseradish peroxidase (HRP). Employing this antigen, two serological tests, a μ-capture ELISA for the detection of CCHFV-specific IgM antibodies (BLACKBOX CCHFV IgM) and an IgG immune complex (IC) ELISA for the detection of CCHFV-specific IgG antibodies (BLACKBOX CCHFV IgG), were developed. Test performance was evaluated and compared with both in-house gold standard testing by IgM/IgG indirect immunofluorescence (IIF) and commercially available ELISA tests (VectoCrimean-CHF-IgM/IgG, Vector-Best, Russia) using a serum panel comprising paired samples collected in Kosovo during the years 2013–2016 from 15 patients with an acute, RT-PCR-confirmed CCHFV infection, and 12 follow-up sera of the same patients collected approximately one year after having overcome the infection. Reliably detecting IgM antibodies in all acute phase sera collected later than day 4 after onset of symptoms, both IgM ELISAs displayed excellent diagnostic and analytical sensitivity (100%, 95% confidence interval (CI): 85.2%–100.0%). While both IgG ELISAs readily detected the high IgG titers present in convalescent patients approximately one year after having overcome the infection (sensitivity 100%, 95% CI: 73.5%–100.0%), the newly developed BLACKBOX CCHFV IgG ELISA was superior to the commercial IgG ELISA in detecting the rising IgG titers during the acute phase of the disease. While all samples collected between day 11 and 19 after onset of symptoms tested positive in both the in-house gold standard IIFT and the BLACKBOX CCHFV IgG ELISA (sensitivity 100%, 95% CI: 71.5%–100.0%), only 27% (95% CI: 6.0%–61.0%) of those samples were tested positive in the commercial IgG ELISA. No false positive signals were observed in either IgM/IgG ELISA when analyzing a priori CCHFV IgM/IgG negative serum samples from healthy blood donors, malaria patients and flavivirus infected patients as well as CCHFV IgM/IgG IIFT negative serum samples from healthy Kosovar blood donors (for BLACKBOX CCHFV IgM/IgG: n = 218, 100% specificity, 95% CI: 98.3%–100.0%, for VectoCrimean-CHF-IgM/IgG: n = 113, 100% specificity, 95% CI: 96.8%–100.0%).

## Author summary

Being endemic in several countries in Asia, Africa, the Middle East and Southeastern Europe, the Crimean-Congo Hemorrhagic Fever Virus (CCHFV) is the geographically most widespread tick-borne arbovirus. As evidenced by the recent occurrence of an autochthonous CCHFV infection in Spain, it possesses also a significant potential to spread to as yet non-endemic regions. Due to the severity of the disease caused by this bunyavirus, the lack of specific prophylactic and therapeutic measures and the infection’s epidemic potential, CCHFV was included in the WHO priority list of diseases needing urgent R&D attention, in particular the development and improvement of diagnostic tools. Here we present the development and validation of two novel ELISAs (BLACKBOX CCHFV IgM, BLACKBOX CCHFV IgG) for the detection of CCHFV-specific IgM and IgG antibodies employing recombinant CCHFV nucleoprotein (NP) as antigen. Test performance in comparison to both in-house gold standard testing (CCHFV IgM/IgG immunofluorescence test (IIFT)) and commercial ELISA kits (VectoCrimean-CHF-IgM/IgG; Vector-Best) was evaluated using a thoroughly characterized serum panel that was obtained from 15 Kosovar patients with an RT-PCR-confirmed CCHFV-infection collected during the years 2013–2016 and that comprised samples from both the acute and convalescent phase of the disease. While both IgM ELISAs, like the CCHFV IgM IIFT, detected CCHFV-specific IgM antibodies in all sera collected during the acute phase of the disease on day 5 after onset of symptoms or later, the BLACKBOX CCHFV IgG ELISA and the CCHFV IgG IIFT were found to be significantly more sensitive than the VectoCrimean-CHF-IgG ELISA in detecting the rising IgG antibody titers in samples collected between days 11 and 19 after onset of symptoms.

## Introduction

Being endemic in a variety of countries in Asia, Africa, the Middle East and Southeastern Europe [1], the Crimean-Congo Hemorrhagic Fever Virus (CCHFV, family: *Nairoviridae*, genus: *Orthonairovirus*; [2]) is the geographically most widespread tick-borne pathogen [3,4]. An infection with this zoonotic virus occurs either by tick bite or contact with body fluids or tissues of viremic humans or animals [5]. After an incubation time of 1–9 days, unspecific flu-like symptoms develop during the first week of disease including high fever, myalgia, arthralgia and headache [5]. Following this prehemorrhagic phase, the disease may progress to the hemorrhagic phase manifesting in petechiae, hematomas, gastrointestinal bleeding (hematemesis, melena), urinary tract bleeding (hematuria) and/or respiratory tract bleeding (hemoptysis) [6]. Case fatality rates (CFRs) range widely between 5% and more than 50% [6] with decreased platelet counts, elevated aspartate transferase and alanine transferase and decreased fribrinogen levels being predictive of fatal outcome [7]. In addition, no CCHFV-specific humoral immune response is observable in the majority of fatal cases [7,8].

CCHF affects residents of endemic countries but can also be imported into non-endemic areas by travelers [1]. Furthermore, the geographic distribution of the disease is expanding as evidenced by the prevalence of CCHFV-infected ticks [9] and the recent occurrence of autochthonous CCHF in Spain [10]. Up to now, neither a vaccine with the potential to gain widespread international regulatory approval nor a specific treatment is available [4], which restricts prophylaxis to exposure prevention and therapy to mainly supportive measures.

Due to the severity of the disease, the lack of specific prophylactic and therapeutic options and its epidemic potential, CCHFV was included in the 2015 WHO priority list of diseases needing urgent R&D attention [11], in particular the development and improvement of diagnostic tools. Although the specificity of commercially available serological assays for the detection of CCHFV-specific antibodies in human sera was found to be excellent in a collaborative evaluation of CCHF diagnostic tests [3], the reported assay sensitivities of 87.8% (range 75.2%–95.3%) for IgM ELISA testing and 80% (range 66.9%–90.2%) for IgG ELISA testing [3] left room for improvements.

Therefore, we developed and validated both a CCHFV μ-capture ELISA (BLACKBOX CCHFV IgM ELISA) and a CCHFV IgG immune complex (IC) ELISA (BLACKBOX CCHFV IgG ELISA) based on the directly horseradish-peroxidase (HRP) labeled, recombinantly produced CCHFV ortholog of the major bunyavirus antigen [12], the nucleoprotein (NP). Assay performance was evaluated in comparison with in-house gold standard testing by IgM/IgG indirect immunofluorescence (IIF) and commercially available IgM/IgG ELISA tests (VectoCrimean-CHF-IgM/IgG, Vector-Best, Russia) using a serum panel comprising paired samples from 15 Kosovar patients with an RT-PCR-confirmed acute CCHFV-infection and 12 follow-up samples from the same group of patients taken approximately one year after they had overcome the disease.

## Materials and methods

### Generation of prokaryotic expression vectors

The plasmid pOPINJ-CCHFV-NP was kindly provided by Sophia Reindl (Department of Virology, BNITM). Briefly, a cDNA fragment encoding the nucleoprotein (NP) of CCHFV strain Afg09-2990 (ADQ57288, [13]) was cloned into the prokaryotic expression vector pOPINJ [14]. The resulting plasmid pOPINJ-CCHFV-NP encodes a 82 kDa fusion protein comprising an N-terminal 6 x His-Tag followed by Glutathione-S-transferase (GST; 26 kDa), a 3C protease cleavage site and the CCHFV-NP (54 kDa).

### Recombinant expression, purification and HRP-labeling of CCHFV-NP

**Expression in *E*.*coli*:** The plasmid pOPINJ-CCHFV-NP was transformed into *E*.*coli* pAPlacI^Q^ cells. Cells were grown at 37°C to an optical density at 600 nm of 0.5–0.6 in Luria-Bertani (LB) medium supplemented with kanamycin and ampicillin, then expression of the CCHFV-NP fusion protein was induced by the addition of isopropyl-β-D-thiogalactosid (IPTG). Bacteria were further incubated over night at 18°C and then harvested by centrifugation. **Cell lysis:** Cells were resuspended in lysis buffer (50 mM NaH_2_PO_4_ pH 8.0, 300 mM NaCl, 10 mM imidazole) supplemented with phenylmethylsulfonylfluorid (PMSF) and lysozyme and incubated at 4°C for 30 min before sonification. After sonification, DNAse was added to a final concentration of 10 μg/ml. The lysate was incubated for 15 min on ice and then centrifuged at 10000 x g for 20 min at 4°C. **Ni-NTA chromatography (native conditions):** After centrifugation of the lysate, the resulting supernatant was incubated with pre-equilibrated Ni-NTA-agarose (Qiagen) for 1 h at 4°C. The matrix was transferred to a column and the flow-through was discarded. After washing the column, bound protein was eluted with Elution Buffer (50 mM NaH_2_PO_4_ pH 8.0, 300 mM NaCl, 250 mM imidazole). **On-column-cleavage of His**_**6**_**-GST tandem tag:** The main fractions were pooled and the buffer was exchanged to Binding Buffer (50 mM NaH_2_PO_4_ pH 7.2, 300 mM NaCl, 5 mM dithiothreitol (DTT)) using Zeba Spin Desalting Columns (Thermo Scientific). Subsequently, tagged recombinant CCHFV-NP was coupled to pre-equilibrated Glutathione HiCap matrix (Qiagen). Unbound material was removed from the column by washing with Binding Buffer. Recombinant GST-tagged 3C-protease was homogenously added to the column matrix and cleavage was performed over night at 4°C with intermittent gentle shaking. Subsequently, CCHFV-NP was eluted from the column and concentrated using Amicon Ultra 15 micro concentrators (Merck Millipore, cut-off: 10k). **Size exclusion chromatography (SEC):** The eluted, concentrated protein was further purified by SEC using a Superdex 200 10/300 GL-column on an ÄKTA pure chromatography system (GE Healthcare). Peak fractions containing recombinant CCHFV-NP were pooled and concentrated using Amicon Ultra 15 micro concentrators (cut-off: 10k). **HRP-labeling:** For labeling recombinant CCHFV-NP with HRP, HRP (Sigma) was activated for coupling with sodium periodate (final concentration 3.5 mg/ml) for 20 min at room temperature (RT). Subsequently, dialysis against 1 mM acetate buffer pH 4.4 was performed at 4°C over night. Activated HRP was incubated with recombinant CCHFV-NP for 4 h at 4°C and sodium borhydride was added at a final concentration of 0.2 mg/ml. After a 5 min incubation at RT, the labeling reaction mix was diluted 1:10 with Freezing Buffer (0.5 x phosphate-buffered saline (PBS), 1% bovine serum albumin (BSA), 0.5% fetal calf serum (FCS), 1% Nonidet P40, 50% glycerol) and stored at - 20°C.

### Enzyme-linked immunosorbent assays (ELISAs)

**IgM μ-capture ELISA (BLACKBOX CCHFV IgM):** ELISA plates (MaxiSorp, Nunc) were coated over night at 4°C with 4 μg/ml goat anti-human IgM (KPL) in PBS pH 7.4. After washing the plates two times with Blocking Buffer (PBS pH 7.4, 0.25% BSA, 0.05% Tween 20), plates were blocked with Blocking Buffer for 2 h at RT. Plates were washed two times with PBS pH 7.4, stabilized and dried using a commercial plate stabilizer (Liquid PlateSealer, Candor) according to the manufacturer’s recommendations. For detection of anti-CCHFV-NP IgM antibodies in human sera, serum samples were diluted 1:100 in Serum Dilution Buffer (PBS pH 7.4, 0.05% Proclin 300, 0.01% phenol red). Per well, 50 μl of diluted serum was applied and the plates were incubated for 60 min at RT (23°C). After washing the plates three times with Wash Buffer (100 mM Tris/HCl pH 7.4, 150 mM NaCl, 0.05% Tween 20, 0.005% ProClin 300), 300 μl/well), 50 μl HRP-labeled CCHFV-NP (final dilution 1:50,000 in Conjugate Dilution Buffer (PBS pH 7.4, 1% BSA, 0.5% FCS, 1% Nonidet P40, 0.1% ProClin 300)) was added per well and the plates were incubated for 60 min at RT (23°C). Plates were washed again three times with Wash Buffer (300 μl/well), and 100 μl SureBlue Reserve TMB Microwell Peroxidase Substrate (KPL) was added per well. Plates were incubated for 20 min at RT (23°C) and the reaction was then stopped by the addition of 100 μl 1N sulfuric acid (Merck) per well. The HRP reaction product was quantified by measuring optical density at 450 nm and 620 nm on a Spectrostar Nano ELISA reader (BMG Labtech).

**IgG Immune-Complex (IC) ELISA (BLACKBOX CCHFV IgG):** The extracellular part of human CD32 (FcγRIIa H131) was recombinantly expressed *in E*.*coli* and purified basically as described previously [15]. ELISA plates (MaxiSorp, Nunc) were coated at 4°C with 5 μg/ml recombinant CD32 protein in Coating Buffer (PBS pH 7.4, 0.01% NaN_3_, 0.01% phenol red). After washing three times with 100 mM Tris pH 7.4, 150 mM NaCl, plates were stabilized and dried using a commercial plate stabilizer (Liquid PlateSealer, Candor) according to the manufacturer’s recommendations. For detection of anti-CCHFV-NP antibodies in human sera, serum samples were diluted 1:50 in Serum Dilution Buffer (PBS pH 7.4, 0.05% Proclin 300, 0.01% phenol red). Per well, 25 μl of diluted human serum were mixed with 25 μl HRP-labeled CCHFV-NP diluted 1:125,000 in Conjugate Dilution Buffer (PBS pH 7.4, 1% BSA, 0.5% FCS, 1% Nonidet P40, 0.1% ProClin 300); final concentrations in well: serum 1:100, HRP-labeled CCHFV-NP 1:250,000. Plates were sealed with adhesive foil and incubated for 24 h at 4°C. Plates were washed three times with Wash Buffer (100 mM Tris/HCl pH 7.4, 150 mM NaCl, 0.05% Tween 20, 0.005% ProClin 300; 300 μl/well)) and 100 μl SureBlue Reserve TMB Microwell Peroxidase Substrate (KPL) was added per well. Plates were incubated for 20 min at RT (23°C) and the reaction was then stopped by the addition of 100 μl 1 N sulfuric acid (Merck) per well. The HRP reaction product was quantified by measuring optical density at 450 nm and 620 nm on a Spectrostar Nano ELISA reader (BMG Labtech).

The IgG immune complex technology employing recombinant CD32 is protected by European (EP2492689) and international (CN103460048, HK1192320, CA2823107, US2014080120) patents owned by the Bernhard Nocht Institute for Tropical Medicine (BNITM).

**VectoCrimean-CHF-IgM/IgG ELISA (Vector-Best, Koltsovo, Russia):** assays (IgM: μ-capture ELISA, IgG: indirect ELISA using immobilized CCHFV antigen) were performed and evaluated according to the manufacturer’s instructions.

### Data analysis

Statistical analysis (One-way ANOVA/Tukey’s multiple comparison test, Fisher’s exact test, calculation of 95% confidence intervals) was performed with GraphPad Prism. Receiver operating characteristic (ROC) curves were generated using MedCalc.

### Sequence analyses

S segment nucleotide sequences of CCHFV strains were downloaded from the National Center for Biotechnology Information (NCBI) Nucleotide database. Alignments of derived protein sequences and generation of identity/similarity matrices were performed with the ClustalW alignment tool of the MacVector (version 12.7.5) software.

### Human sera

**CCHF patient sera:** Paired serum samples were obtained from 15 Kosovar patients with a PCR-confirmed CCHFV-infection (13/15 (87%) male; 2/15 (13%) female; median age: 40 years (range 10–75) between 2013 and 2015 (2013: n = 10, 2014: n = 4, 2015: n = 1) in the course of the joint research project (BNITM, University of “Hasan Prishtina” and National Institute of Public Health, Pristhina, Kosovo) “Diagnosis and surveillance of Crimean-Congo hemorrhagic fever (CCHF) in Kosovo”, funded by the German Ministry of Foreign Affairs (Project-No 68777 EN 02761868). Eight of 15 (53%) patients had a known history of tick bite (onset of symptoms after tick bite median 2.5 days (range 1–5 days)). From each of the 15 patients, one “early” sample collected between day 2 and day 14 after onset of symptoms (median: 5 days) and one “late” sample collected 3 to 23 days after the “early” sample (median: 10 days) between day 8 and day 36 after onset of symptoms (median: 17 days) were analyzed. CCHFV-infection has been proven by RT-PCR (RealStar CCHFV RT PCR Kit, Altona Diagnostics) for all 15 patients in a serum sample collected directly after admission to the hospital. Nevertheless, for five patients this sample was not available for ELISA testing due to scarcity of material. Thus, for all patients, the earliest available serum sample (which may or may not be PCR positive) was chosen as “early” sample for the ELISA analysis. In addition, “convalescent” samples were obtained from 12 patients approximately one year after recovery from CCHFV infection. IgM/IgG status of patient samples was characterized by in-house IgM/IgG IIFT using acetone-fixed Vero cells infected with CCHFV strain ArD39554 as described previously [16]. Sera were inactivated by the addition of Triton X-100 to a final concentration of 1% prior to serological testing.

**Sera from healthy blood donors from Kosovo:** Sera were obtained from 98 healthy donors (77/98 (79%) male; 21/98 (21%) female; median age: 33 years (range 20–60)). All sera had previously been tested negative by in-house CCHFV IgM/IgG IIFT.

### A priori CCHFV IgM/IgG negative control sera

**DENV:** 8 DENV IgM/IgG positive sera were obtained from European travelers with a PCR confirmed DENV infection (2 x DENV1, 2 x DENV2, 2 x DENV3, 2 x DENV4).

**TBEV:** 2 TBEV IgM/IgG positive sera were obtained from Biomex (Germany).

**Malaria:** 27 sera from patients with an acute *P*. *falciparum* malaria (confirmed by IIFT) and 6 sera from patients with an acute *P*. *malariae* infection (confirmed by IIFT) were obtained from the BNITM diagnostics department (section parasitology).

**Healthy blood donors (HD): HD Europe:** 49 sera from healthy blood donors from Europe were taken at the BNITM between the years 2012 and 2016. **HD Asia/HD South America:** Sera of healthy blood donors from Lao PDR (n = 14) and Colombia (n = 14) were collected during the projects “Savannakhet-Hamburg Research Program on Neglected Diseases” and “Valledupar-Hamburg research programme on diagnostics and research on tropical and emerging infections” in 2012 and 2013, respectively. Healthy blood donors from Colombia had been vaccinated against YF.

### Ethics statement

The study complies with the Declaration of Helsinki. Written informed consent was obtained from all individuals or, in case of minors, from parents or legal guardians before enrollment. Data privacy protection was guaranteed by anonymization of serum samples.

Collection of serum samples was approved by the Ethics Committee of the University of Prishtina “Hasan Pristhina” (sera from CCHF patients and healthy blood donors from Kosovo), the Ethics Committee of the Lao People’s Democratic Republic (sera from healthy blood donors from Lao PDR, no. 030/NECHR), the Ethics Committee of the Hospital Rosario Pumarejo de Lopez of Valledupar/Colombia (sera from healthy blood donors from Colombia) and the Ethics Committee of the Ärztekammer Hamburg (sera from Dengue fever patients, malaria patients and healthy blood donors from EU, no. PV4608).

## Results

### Recombinant expression and purification of CCHFV-NP

**Fig 1:**
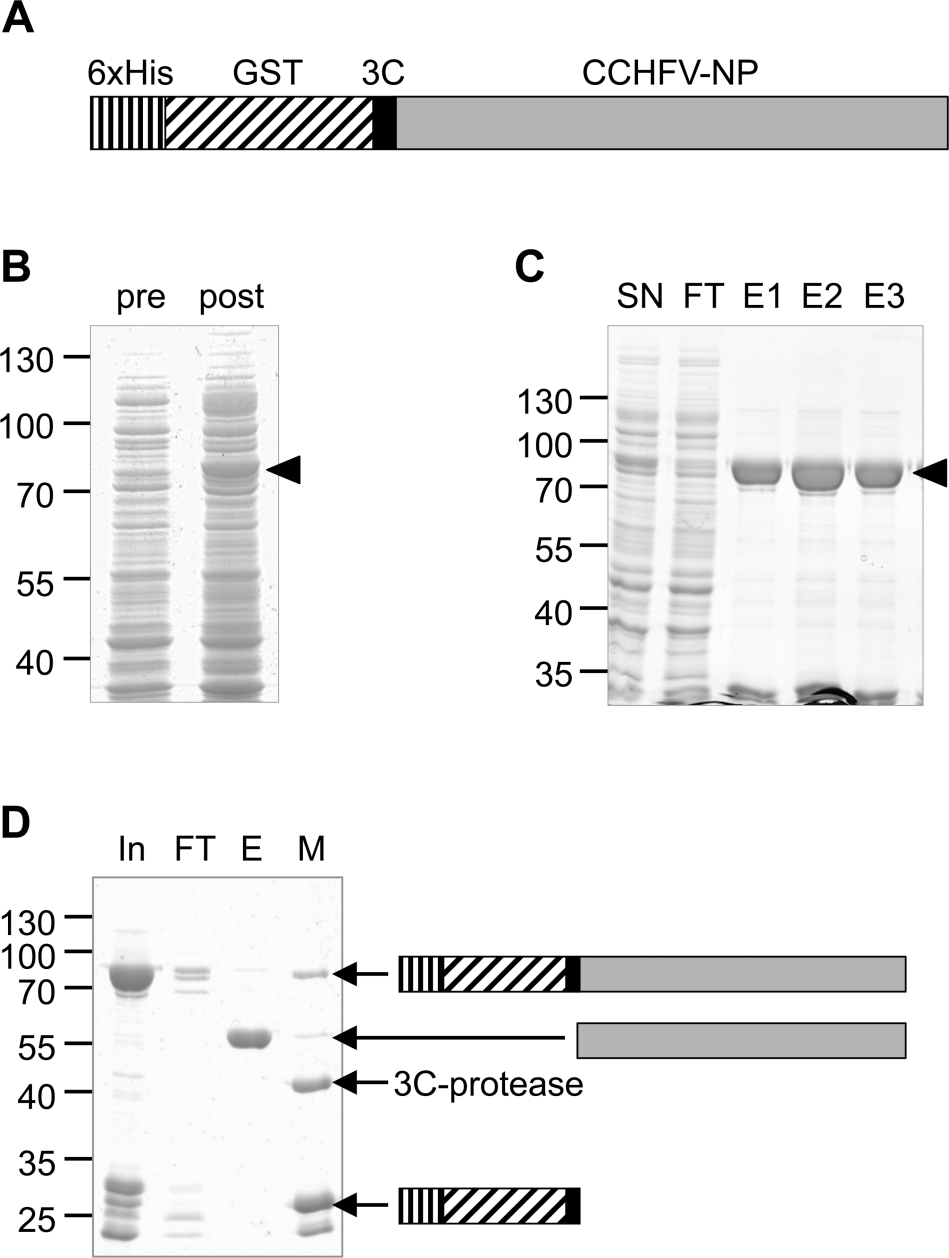
Prokaryotic expression and purification of CCHFV-NP. **(A) Primary structure of recombinant CCHFV-NP fusion protein** encoded by the expression vector pOPINJ-CCHFV-NP (6xHis-GST-3C-CCHFV-NP). The full-length CCHFV-NP is N-terminally tagged with both a 6 x His-tag and GST. A 3C protease recognition site enables tag removal after purification. **(B) Induction of protein expression by IPTG**. Protein expression was induced by IPTG in *E*.*coli* pAPlacI^Q^ cells transformed with pOPINJ-CCHFV-NP. Total bacterial lysates (pre: before induction, post: after induction) were analyzed by SDS-PAGE followed by Coomassie-staining. Arrow head: 6xHis-GST-3C-CCHFV-NP (calculated molecular weight: 82 kDa). **(C) Ni-NTA affinity purification** of 6xHis-GST-3C-CCHFV-NP under native conditions. *E*.*coli* pAPlacI^Q^ harbouring 6xHis-GST-3C-CCHFV-NP were lyzed by lysozyme and sonification. After centrifugation, the soluble supernatant (SN) was applied to Ni-NTA agarose. Bound material was eluted by imidazole. FT: flow through, eluates: E1, E2, E3. Arrow head: 6xHis-GST-3C-CCHFV-NP (calculated molecular weight: 82 kDa). **(D) Removal of 6xHis-GST tag** by on-column 3C protease digest. Eluates from Ni-NTA affinity purification were pooled (In: Input) and applied to Glutathion HiCap matrix (FT: Flow-through). On-column cleavage was performed using recombinant, GST-tagged 3C-protease. E: Eluate from matrix (CCHFV-NP without tag, calculated molecular weight: 54 kDa), M: Proteins attached to the matrix after elution.

In order to generate highly purified recombinant CCHFV-NP for use as an antigen in CCHFV ELISA applications a fusion protein consisting of an N-terminal His/GST-tandem tag and the full length CCHFV-NP separated by a 3C protease cleavage site (**Fig 1A**) was expressed in *E*.*coli* (**Fig 1B**). The recombinant fusion protein was purified from the soluble fraction of the bacterial lysate by Ni-NTA affinity chromatography (**Fig 1C**). After removal of the tandem tag with 3C protease by on-column cleavage on glutathione matrix (**Fig 1D**), CCHFV-NP was further purified by size exclusion chromatography and directly labeled with horseradish-peroxidase (HRP).

### Development of IgM/IgG ELISA protocols using HRP-labeled recombinant CCHFV-NP as antigen

Using the HRP-labeled recombinant CCHFV-NP (CCHFV-NP^HRP^) as antigen, both a μ-capture ELISA protocol for the detection of human anti-CCHFV IgM antibodies (designated as “BLACKBOX CCHFV IgM”) and an IgG IC ELISA protocol for the detection of human anti-CCHFV IgG antibodies (designated as “BLACKBOX CCHFV IgG”) were developed.

For the BLACKBOX CCHFV IgM ELISA, plates were coated with anti-human IgM antibodies and subsequently incubated for 1 h at RT with human serum and CCHFV-NP^HRP^. After washing, binding was quantified by measuring optical density after incubation with a chromogenic HRP-substrate.

For the BLACKBOX CCHFV IgG ELISA, plates were coated with a recombinant fragment of the human FcγR CD32 [15,17]. Human serum and CCHFV-NP^HRP^ were co-incubated for 24 h at 4°C on the plates. After washing, binding of immune complexes was quantified by measuring optical density after incubation with a chromogenic HRP-substrate.

### Detection range and reproducibility of BLACKBOX CCHFV IgM/IgG ELISAs

**Fig 2:**
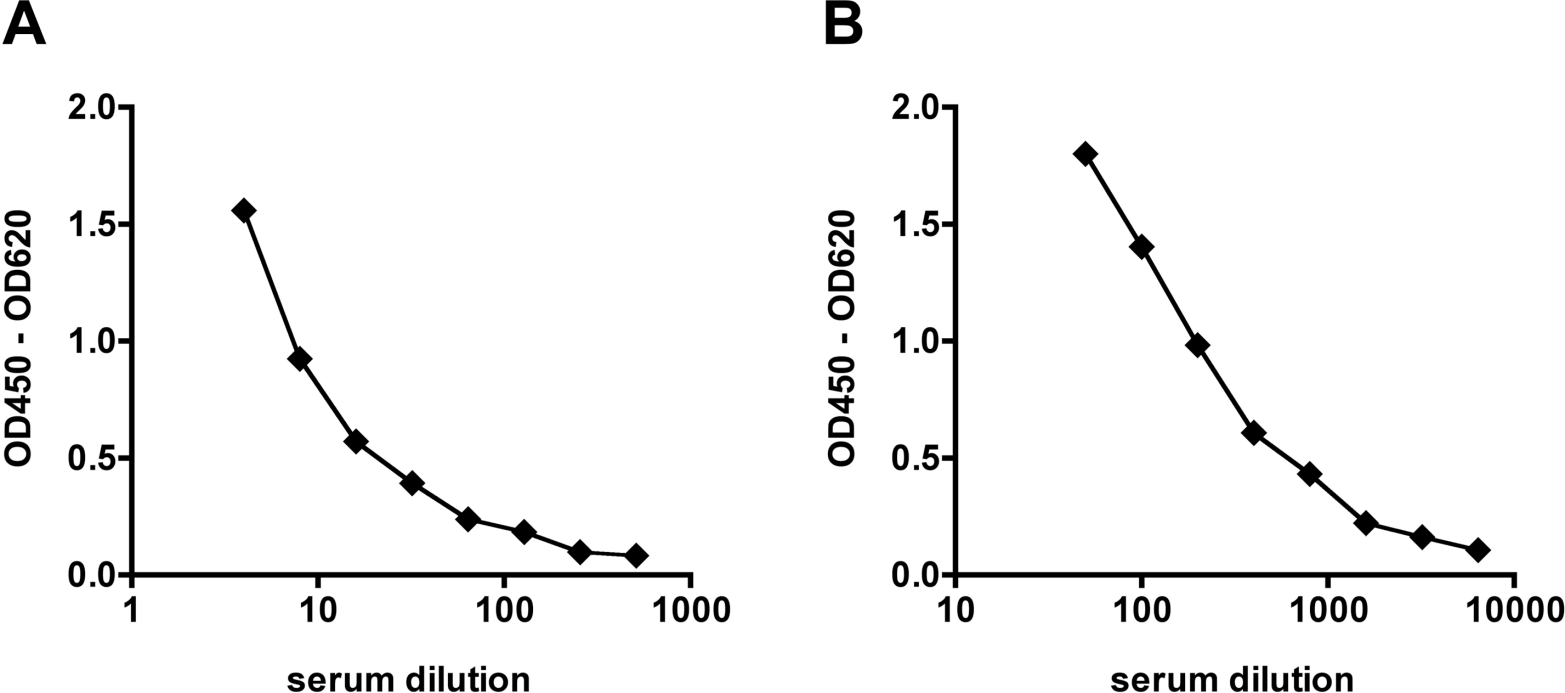
Detection range of BLACKBOX CCHFV IgM/IgG ELISAs. **(A) BLACKBOX CCHFV IgM ELISA.** A CCHF patient serum (IIFT IgM titer 1: 640) was spiked into a negative human serum at different dilutions (1:4, 1:8, 1:16, 1:32, 1:64, 1:128, 1:256, 1:512) and analyzed with the BLACKBOX CCHFV IgM ELISA. In parallel, 71 CCHFV IgM negative sera from healthy human blood donors (n = 49) and malaria infected (n = 22) patients were tested (mean optical density of signals obtained for CCHFV IgM negative sera: 0.032, standard deviation: 0.011). **(B) BLACKBOX CCHFV IgG ELISA**. A CCHF patient serum (IIFT IgG titer 1: 10240) was spiked into a negative human serum at different dilutions (1:50, 1:100, 1:200, 1:400, 1:800, 1:1600, 1:3200, 1:6400) and analyzed with the BLACKBOX CCHFV IgG ELISA. In parallel, 71 CCHFV IgG negative sera from healthy human blood donors (n = 49) and malaria infected (n = 22) patients were tested (mean optical density of signals obtained for CCHFV IgG negative sera: 0.072, standard deviation: 0.017).

Titration of an IgM/IgG IIFT positive patient serum and analysis of a panel of a priori CCHFV IgM/IgG negative sera from healthy human blood donors and malaria patients indicated a broad detection range and low background signal of the assays **(Fig 2)**. Furthermore, intra- and inter-assay variation (**Table 1, Table 2**) as well as inter-laboratory variation (**Table 3, Table 4**) were analyzed, revealing a high reproducibility of both assay systems (mean intra-assay CV for positive sera < 5%; mean inter-assay CV for positive sera < 10%, inter-laboratory CV for positive serum < 10%).

**Table 1:** Intra- and interassay variation of BLACKBOX CCHFV IgM ELISA.

	day 1			day 2			day 3						
sample	mean	sd	CV_intra_	mean	sd	CV_intra_	mean	sd	CV_intra_	CV_intra, av_	mean_av_	sd of mean_av_	CV_inter_
***pos1***	1.076	0.031	2.9	1.179	0.014	1.2	1.179	0.042	3.6	2.5	1.145	0.059	5.2
***pos2***	0.387	0.019	4.9	0.425	0.010	2.2	0.452	0.019	4.3	3.8	0.421	0.033	7.8
***pos3***	0.264	0.012	4.4	0.285	0.004	1.4	0.299	0.009	3.2	3.0	0.283	0.018	6.4
***neg1***	0.054	0.002	3.7	0.054	0.003	4.6	0.074	0.003	4.3	4.2	0.061	0.012	19.1
***neg2***	0.046	0.003	6.9	0.047	0.003	5.4	0.067	0.004	6.1	6.1	0.053	0.012	21.9
***neg3***	0.064	0.002	2.4	0.069	0.002	2.2	0.083	0.007	8.4	4.3	0.072	0.010	13.6
										**mean**_**pos**_**: 3.1**			**mean**_**pos**_**: 6.5**
										**mean**_**neg**_**: 4.9**			**mean**_**neg**_**: 18.2**

To determine the intra- and inter-assay variation of the BLACKBOX CCHFV IgM ELISA, the absolute OD values (OD450 – OD620) for three anti-CCHFV-NP IgM positive samples (pos1, pos2, pos3) and three anti-CCHFV-NP IgM negative samples (neg1, neg2, neg3) were measured in triplicate on three different days (day 1, day 2, day 3). Triplicates were used to calculate the intra-assay coefficient of variation for each individual experiment (CV_intra_, in %), mean CV_intra_ (CV_intra,av_, in %) was calculated by averaging the three obtained CV_intra_ values. The inter-assay coefficient of variation (CV_inter_, in %) was calculated according to the formula CV_inter_ = 100 * (sd of mean_av_)/mean_av_ with mean_av_ being the mean of the mean OD values obtained for the respective sample on day 1, day 2 and day 3 and sd of mean_av_ being the standard deviation of those three values.

**Table 2:** Intra- and interassay variation of BLACKBOX CCHFV IgG ELISA.

	day 1			day 2			day 3						
sample	mean	sd	CV_intra_	mean	sd	CV_intra_	mean	sd	CV_intra_	CV_intra, av_	mean_av_	sd of mean_av_	CV_inter_
***pos1***	1.486	0.045	3.1	1.520	0.067	4.4	1.404	0.057	4.0	3.8	1.470	0.059	4.0
***pos2***	0.534	0.016	3.0	0.522	0.006	1.2	0.514	0.023	4.5	2.9	0.523	0.010	2.0
***pos3***	0.225	0.015	6.6	0.208	0.010	4.9	0.203	0.010	4.7	5.4	0.212	0.011	5.4
***neg1***	0.040	0.003	6.2	0.032	0.001	1.8	0.031	0.002	5.6	4.5	0.035	0.005	14.6
***neg2***	0.045	0.002	3.4	0.037	0.001	3.1	0.034	0.002	5.1	3.9	0.039	0.005	14.1
***neg3***	0.051	0.003	5.0	0.042	0.002	3.7	0.040	0.002	5.7	4.8	0.044	0.006	12.7
										**mean**_**pos**_**: 4.0**			**mean**_**pos**_**: 3.8**
										**mean**_**neg**_**: 4.4**			**mean**_**neg**_**: 13.8**

To determine the intra- and inter-assay variation of the BLACKBOX CCHFV IgG ELISA, the absolute OD values (OD450 – OD620) for three anti-CCHFV-NP IgG positive samples (pos1, pos2, pos3) and three anti-CCHFV-NP IgG negative samples (neg1, neg2, neg3) were measured in triplicate on three different days (day 1, day 2, day 3). Triplicates were used to calculate the intra-assay coefficient of variation for each individual experiment (CV_intra_, in %), mean CV_intra_ (CV_intra,av_, in %) was calculated by averaging the three obtained CV_intra_ values. The inter-assay coefficient of variation (CV_inter_, in %) was calculated according to the formula CV_inter_ = 100 * (sd of mean_av_)/mean_av_ with mean_av_ being the mean of the mean OD values obtained for the respective sample on day 1, day 2 and day 3 and sd of mean_av_ being the standard deviation of those three values.

**Table 3:** Inter-laboratory variation of BLACKBOX CCHFV IgM ELISA.

	positive control sample				negative control sample			
	n	mean	sd	CV_inter_	n	mean	sd	CV_inter_
**laboratory 1**	5	1.446	0.153	10.6	3	0.041	0.005	12.2
**laboratory 2**	5	1.428	0.134	9.4	5	0.031	0.015	48.4
**laboratory 3**	4	1.461	0.187	12.8	4	0.021	0.003	14.3
**mean**		1.445				0.031		
**sd**		0.017				0.010		
**CV**_**inter-lab**_		1.14				32.26		

To determine the inter-laboratory variation of the BLACKBOX CCHFV IgM ELISA, n independent measurements of the absolute OD values (OD450 – OD620) for an anti-CCHFV-NP IgM positive sample and an anti-CCHFV-NP IgM negative sample were performed in three different laboratories. The inter-laboratory coefficient of variation (CV_inter-lab_, in %) was calculated according to the formula CV_inter-lab_ = 100 * sd/mean with mean being the mean of the OD values obtained for the respective sample in the three different laboratories (each as a mean of n independent measurements) and sd being the standard deviation of those three values.

**Table 4:** Inter-laboratory variation of BLACKBOX CCHFV IgG ELISA.

	positive control sample				negative control sample			
	n	mean	sd	CV_inter_	n	mean	sd	CV_inter_
**laboratory 1**	5	1.859	0.152	8.2	3	0.053	0.011	20.8
**laboratory 2**	2	2.122	0.134	6.3	2	0.046	0.002	4.3
**laboratory 3**	4	2.216	0.31	14.0	4	0.026	0.005	19.2
**mean**		2.066				0.042		
**sd**		0.185				0.014		
**CV**_**inter-lab**_		8.96				33.63		

To determine the inter-laboratory variation of the BLACKBOX CCHFV IgG ELISA, n independent measurements of the absolute OD values (OD450 – OD620) for an anti-CCHFV-NP IgG positive sample and an anti-CCHFV-NP IgG negative sample were performed in three different laboratories. The inter-laboratory coefficient of variation (CV_inter-lab_, in %) was calculated according to the formula CV_inter-lab_ = 100 * sd/mean with mean being the mean of the OD values obtained for the respective sample in the three different laboratories (each as a mean of n independent measurements) and sd being the standard deviation of those three values.

### Determination of optimal assay cut-offs for the BLACKBOX CCHFV IgM ELISA and the BLACKBOX CCHFV IgG ELISA, assay sensitivities and specificities

**Fig 3:**
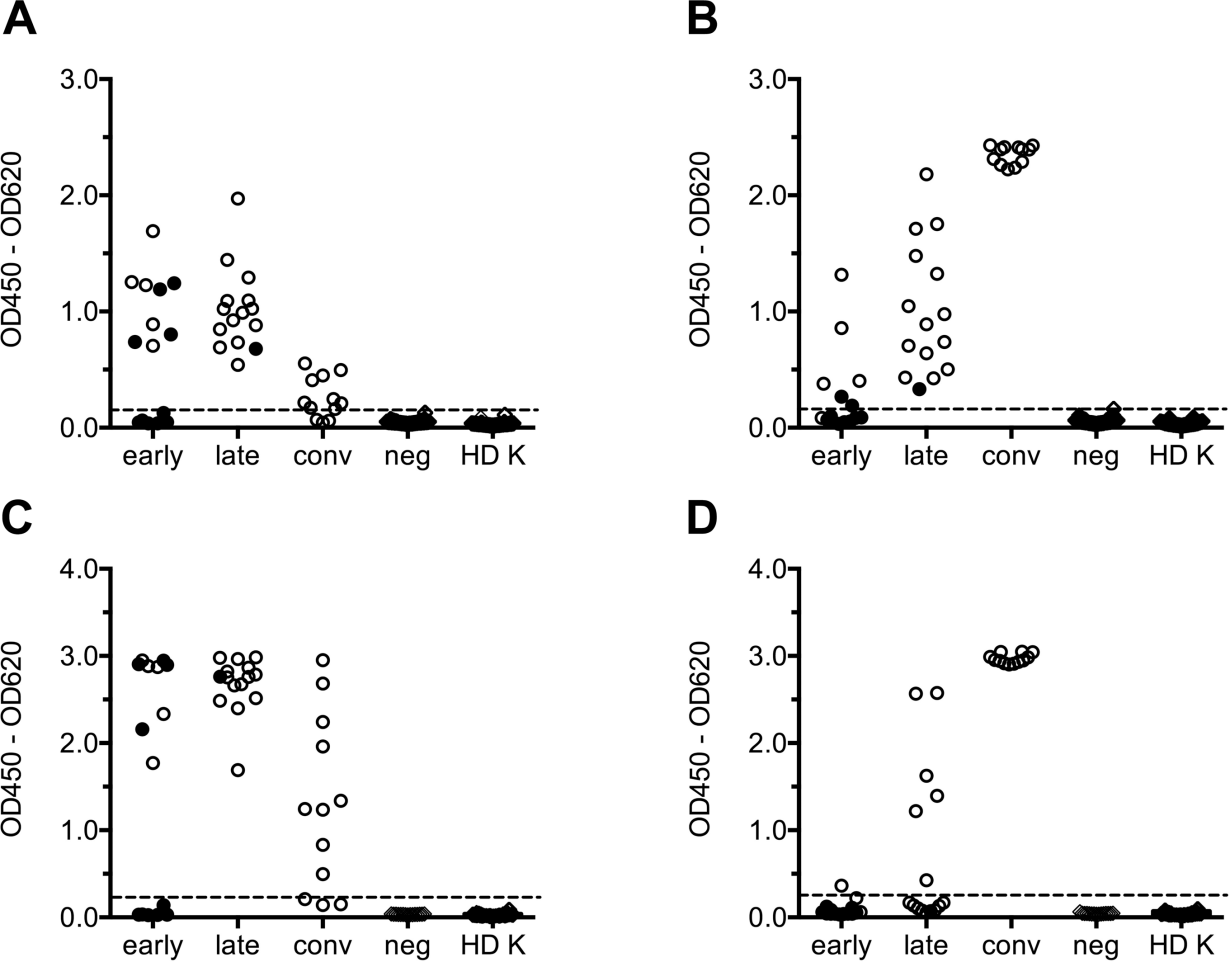
Analysis of paired CCHF patient samples: Raw data (OD420 – OD620). A serum panel consisting of 30 paired serum samples from 15 CCHF patients, serum samples from 12 CCHF patients collected approximately one year after recovery from CCHFV infection, a set of a priori CCHFV IgM/IgG negative serum samples (neg) ((A), (B): n = 120; (C), (D): n = 15, see S1 Fig), and 98 CCHFV IgM/IgG IIFT negative sera from healthy blood donors from Kosovo (HD K) were analyzed with the **BLACKBOX CCHFV IgM ELISA (A)**, the **BLACKBOX CCHFV IgG ELISA (B),** the **VectoCrimean-CHF-IgM ELISA (C)** and the **VectoCrimean-CHF-IgG ELISA (D).** Cut-off values (represented by dotted lines) were determined by ROC analysis ((A): 0.129, (B): 0.161) or according to the manufacturer’s instructions ((C): 0.240, (D): 0.246), respectively (see S2 Fig). Filled circles indicate PCR positive serum samples.

For assay validation, a serum panel comprising serum samples from 15 CCHF patients from Kosovo (**Table 5**) was analyzed with the BLACKBOX CCHFV IgM ELISA (**Fig 3A**) and the BLACKBOX CCHFV IgG ELISA (**Fig 3B**). From each of the 15 patients, one “early” sample collected between day 2 and day 14 after onset of symptoms (median: 5 days) and one “late” sample collected 3 to 23 days after the “early” sample (median: 10 days) between day 8 and day 36 after onset of symptoms (median: 17 days) were tested. In addition, “convalescent” samples taken from 12 patients approximately one year after recovery from CCHFV infection were analyzed. To evaluate assay specificity, 120 a priori CCHFV IgM/IgG negative sera originating from either healthy blood donors from Germany (Europe), Colombia (South America) and Lao PDR (Asia) (n = 77), malaria patients (n = 33) or flavivirus infected patients (n = 10) were tested along with 98 CCHFV IgM/IgG IIFT negative sera from healthy blood donors from Kosovo (**Fig 3A, Fig 3B, panels A and B in S1 Fig**).

**Table 5:** CCHF patient sera information and results summary.

PATIENT				„EARLY‟SAMPLE								„LATE‟SAMPLE								„CONV‟SAMPLE							
no.	age	gender	tick bite	d.a.o.	PCR	IgM			IgG			d.a.o.	PCR	IgM			IgG			d.a.o.	PCR	IgM			IgG		
						IIFT	BB	VB	IIFT	BB	VB			IIFT	BB	VB	IIFT	BB	VB			IIFT	BB	VB	IIFT	BB	VB
**1**	10	m	yes	6	pos	pos	pos	pos	pos	pos	neg	15	neg	pos	pos	pos	pos	pos	neg	> 1 year	nt	neg	neg	neg	pos	pos	pos
**2**	75	m	yes	14	amb	pos	pos	pos	pos	pos	ambig	17	neg	pos	pos	pos	pos	pos	neg	> 1 year	nt	pos	pos	pos	pos	pos	pos
**3**	29	f	yes	13	neg	pos	pos	pos	pos	pos	pos	36	neg	pos	pos	pos	pos	pos	pos	> 1 year	nt	neg	pos	pos	pos	pos	pos
**4**	13	m	yes	2	pos	neg	neg	neg	pos*	neg	neg	11	neg	pos	pos	pos	pos	pos	neg	> 1 year	nt	pos*	pos	pos	pos	pos	pos
**5**	41	m		8	nt	pos	pos	pos	pos	neg	neg	21	neg	pos	pos	pos	pos	pos	pos	> 1 year	nt	neg	neg	neg	pos	pos	pos
**6**	62	m		3	pos	neg	neg	neg	neg	neg	neg	25	neg	pos	pos	pos	pos	pos	pos								
**7**	54	m		2	pos	neg	neg	neg	neg	neg	neg	8	pos	pos	pos	pos	pos	pos	neg	> 1 year	nt	pos	pos	pos	pos	pos	pos
**8**	12	m		5	pos	pos	pos	pos	pos	pos	neg	15	neg	pos	pos	pos	pos	pos	pos	> 1 year	nt	neg	pos	pos	pos	pos	pos
**9**	70	m	yes	5	pos	pos	pos	pos	pos	neg	neg	12	neg	pos	pos	pos	pos	pos	pos								
**10**	69	m		7	amb	pos	pos	pos	pos	pos	neg	26	nt	pos	pos	pos	pos	pos	neg								
**11**	38	m	yes	4	pos	neg	neg	neg	neg	neg	neg	11	nt	pos	pos	pos	pos	pos	neg	> 1 year	nt	neg	pos	pos	pos	pos	pos
**12**	16	m		7	pos	pos	pos	pos	pos	neg	neg	16	neg	pos	pos	pos	pos	pos	neg	> 1 year	nt	neg	pos	pos	pos	pos	pos
**13**	51	m	yes	3	pos	neg	neg	neg	neg	neg	neg	18	nt	pos	pos	pos	pos	pos	neg	> 1 year	nt	neg	neg	neg	pos	pos	pos
**14**	37	f		8	neg	pos	pos	pos	pos	pos	neg	17	nt	pos	pos	pos	pos	pos	neg	> 1 year	nt	pos	pos	pos	pos	pos	pos
**15**	40	m	yes	3	pos	pos*	neg	neg	neg	neg	neg	20	nt	pos	pos	pos	pos	pos	pos	> 1 year	nt	pos*	pos	pos	pos	pos	pos
	median: 40			median: 5								median: 17															

Patient information: gender: m (male), f (female); tick bite: “yes” if patient reported a recent tick bite; Results: d.a.o. (days after onset of symptoms when the sample was taken); IIFT: indirect immunofluorescence test; BB: BLACKBOX; VB: Vector-Best; pos: positive; neg: negative; ambig: ambiguous; nt: not tested; *: weak IgM IIFT titer (1:40)

For determination of the optimal assay cut-offs, ROC analysis was performed using specific subsets of CCHF patient sera corresponding to the respective assay’s intended diagnostic purpose (**S2 Fig**). The main intention of performing an IgM ELISA is the diagnosis of acute infections as early in the course of disease as possible, whereas IgG testing rather becomes relevant at later stages of acute disease progression and for seroprevalence studies on healthy individuals. Therefore, assay cut-offs were optimized for optimal differentiation of PCR negative “early” and “late” samples from the a priori negative control samples for the BLACKBOX CCHFV IgM ELISA (**panel A in S2 Fig,** cut-off: 0.129) and PCR negative “late” and “convalescent” samples from the a priori negative control samples for the BLACKBOX CCHFV IgG ELISA (**panel B in S2 Fig,** cut-off: 0.161), respectively.

When applying these cut-offs to the complete tested serum panel, both the BLACKBOX CCHFV IgM ELISA (**Fig 3A, Table 6**) and the BLACKBOX CCHFV IgG ELISA (**Fig 3B, Table 7**) display a 100% specificity (95% CI: 98.3%–100.0%) and a 100% sensitivity (95% CI: 78.2%–100.0%) when testing “late” serum samples. While all five PCR negative “early” samples tested positive in the BLACKBOX CCHFV IgM ELISA, CCHFV-specific IgM antibodies could only be detected in 40% of the PCR positive “early” samples (**Fig 3A, Table 6**). The BLACKBOX CCHFV IgG ELISA detected CCHFV-specific antibodies weakly in 2 out of 10 PCR positive “early” samples, but generated a clearly positive result in four out of five PCR negative “early” samples (**Fig 3B, Table 7**). For all 6 patients whose PCR positive “early” samples tested negative in both the BLACKBOX CCHFV IgM ELISA and the BLACKBOX CCHFV IgG ELISA, clear IgM and IgG seroconversion was observed from the “early” to the “late” sample (**panels A and B in S3 Fig**).

**Table 6:** Analysis of paired CCHF patient samples: Comparison of BLACKBOX CCHFV IgM ELISA, VectoCrimean-CHF IgM ELISA and in-house IgM IIFT results.

			IgM tested positive (# (%)) in			results statistical testing		
sample type		# samples	IIFT	BB	VB	IIFT vs. BB	BB vs. VB	VB vs. IIFT
early	PCR pos	10	5 (50)	4 (40)	4 (40)	ns	ns	ns
	PCR amb/neg/nt	5	5 (100)	5 (100)	5 (100)	ns	ns	ns
late		15	15 (100)	15 (100)	15 (100)	ns	ns	ns
conv		12	5 (42)	9 (75)	9 (75)	ns	ns	ns

IIFT: indirect immune fluorescence test, BB: BLACKBOX CCHFV ELISA, VB: VectoCrimean-CHF ELISA. Statistical testing was performed using the Fisher’s exact test routine in GraphPad Prism. Ns: not significant (p > 0.05).

**Table 7:** Analysis of paired CCHF patient samples: Comparison of BLACKBOX CCHFV IgG ELISA, VectoCrimean-CHF IgG ELISA and in-house IgG IIFT results.

			IgG tested positive (# (%)) in			results statistical testing		
sample type		# samples	IIFT	BB	VB	IIFT vs. BB	BB vs. VB	VB vs. IIFT
early	PCR pos	10	5 (50)	2 (20)	0 (0)	ns	ns	*
	PCR amb/neg/nt	5	5 (100)	4 (80)	1 (20)	ns	ns	*
late		15	15 (100)	15 (100)	6 (40)	ns	***	***
conv		12	12 (100)	12 (100)	12 (100)	ns	ns	ns

IIFT: indirect immune fluorescence test, BB: BLACKBOX CCHFV ELISA, VB: VectoCrimean-CHF ELISA. Statistical testing was performed using the Fisher’s exact test routine in GraphPad Prism. Ns: not significant (p > 0.05); *: p < 0.05; ***: p < 0.001.

**Fig 4:**
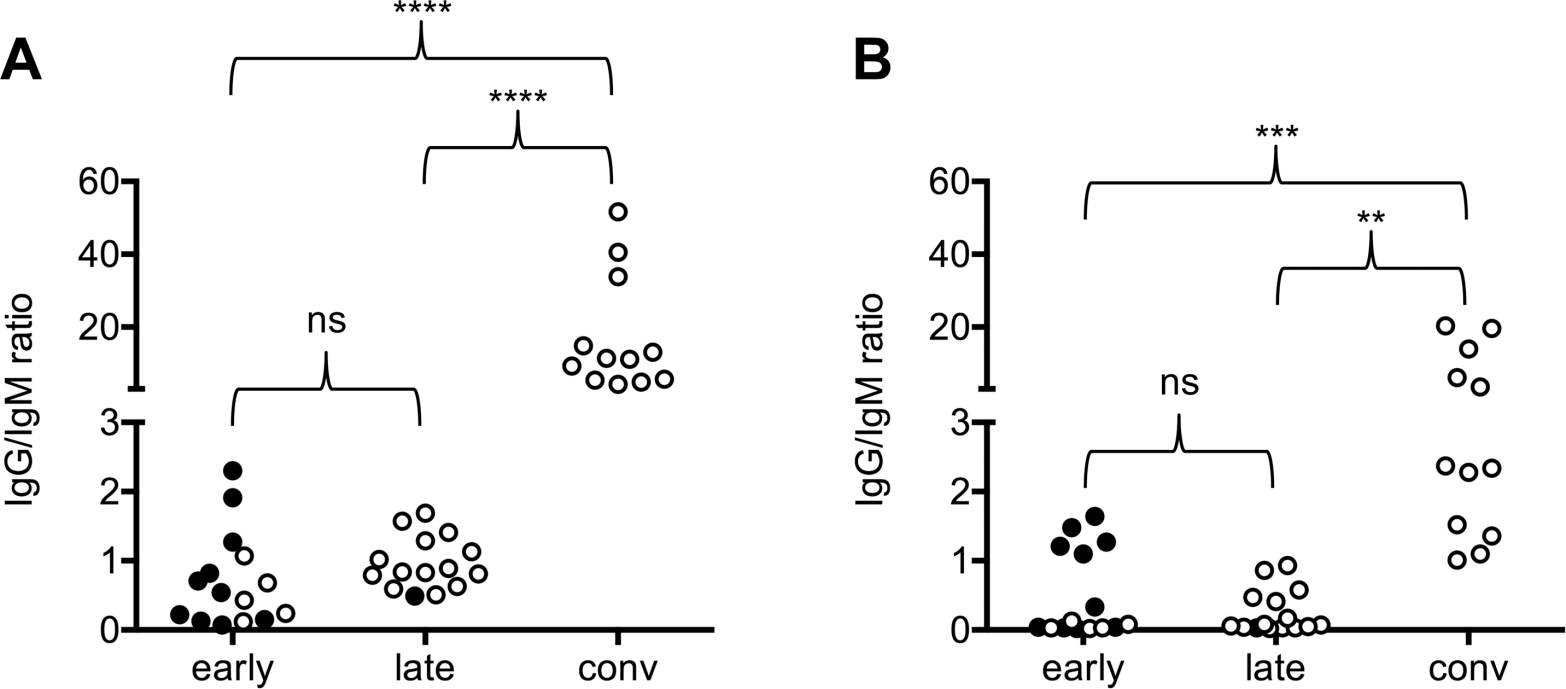
Analysis of paired CCHF patient samples: IgG/IgM ratio. Ratios of OD450 – OD620 values obtained with the **BLACKBOX CCHFV IgG/IgM ELISA (A) and the VectoCrimean-CHF-IgG/IgM ELISA (B)** were calculated for the analyzed serum samples. Filled circles indicate PCR positive serum samples. Statistical testing (One-way ANOVA/Tukey’s multiple comparison test) was performed with GraphPad Prism (ns: not significant (p > 0.05); **: p < 0.01; ***: p < 0.001; ****: p < 0.0001).

Surprisingly, 9 out of 12 “convalescent” serum samples taken from CCHF patients more than one year after having overcome the disease tested weakly positive in the BLACKBOX IgM ELISA (**Fig 3A, Table 6**). Nevertheless, a clear differentiation between an acute CCHFV infection and the convalescent state is possible by calculating the IgG/IgM ratio defined as the quotient of the optical densities obtained when performing the BLACKBOX CCHFV IgG ELISA and the BLACKBOX CCHFV IgM ELISA, respectively (**Fig 4A**). While the IgG/IgM ratio in the patient samples taken during the acute phase of the disease ranges from 0 to < 3.0, the “convalescent” patient samples display a significantly higher IgG/IgM ratio (**Fig 4A**).

### Comparison to in-house gold standard serological testing (CCHFV IgM/IgG IIFT)

All CCHF patient samples had previously been analyzed with CCHFV IgM and CCHFV IgG IIFT (**Table 5**). While the BLACKBOX CCHFV IgM ELISA displayed a comparable sensitivity as the IgM IIFT for all sample types tested (**Table 6**), the CCHFV IgG IIFT detected low IgG titers in 5 of the 10 PCR positive “early” samples while only 2 of those samples were recognized as IgG positive by the BLACKBOX CCHFV IgG ELISA (**Table 7**). Nevertheless, 4 of 5 PCR negative “early” samples as well as all “late” (15/15) and “convalescent” (12/12) samples tested positive in both the CCHFV IgG IIFT and the BLACKBOX CCHFV IgG ELISA (**Table 7**). Thus, the performance of both ELISAs is comparable to gold standard testing when applied according to their respective intended diagnostic purpose (BLACKBOX CCHFV IgM ELISA for acute diagnostics, BLACKBOX CCHFV IgG ELISA for later stages of disease progression and seroprevalence studies).

### Comparison to commercially available ELISA tests (VectoCrimean-CHF-IgM, VectoCrimean-CHF-IgG; Vector-Best)

**Fig 5:**
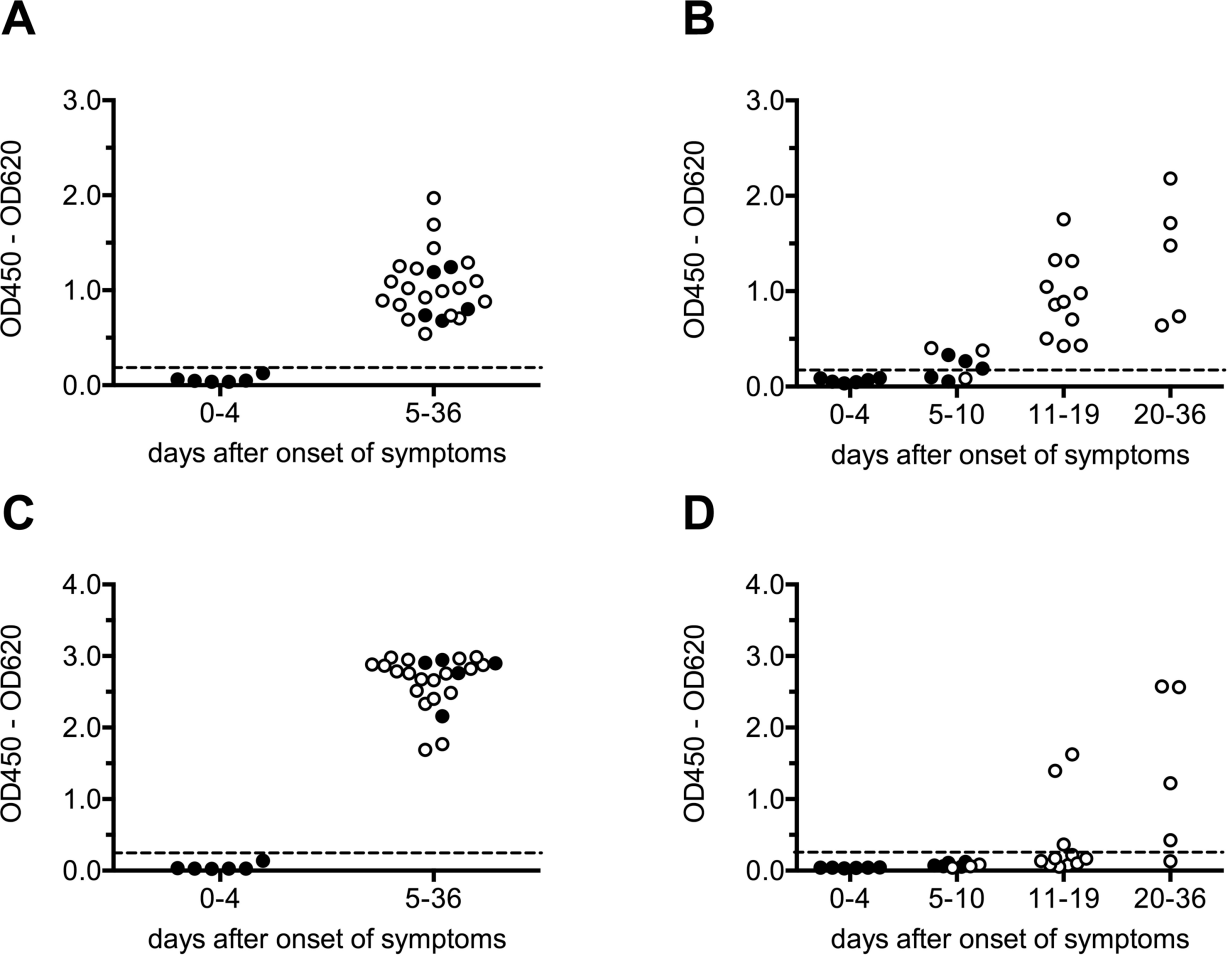
Analysis of CCHF patient samples: Raw data (OD450 – OD620), dependence from day after onset. **(A) BLACKBOX CCHFV IgM ELISA. (B) BLACKBOX CCHFV IgG ELISA. (C) VectoCrimean-CHF-IgM ELISA. (D) VectoCrimean-CHF-IgG ELISA.** Paired serum samples from 15 CCHF patients were classified according to their time point of collection (days after onset of symptoms). Filled circles indicate PCR positive serum samples. Cut-off values (represented by dotted lines) were determined by ROC analysis ((A): 0.129, (B): 0.161) or according to the manufacturer’s instructions ((C): 0.240, (D): 0.246), respectively (see S2 Fig).

The same CCHFV serum panel which had been analyzed with the BLACKBOX CCHFV IgM ELISA and the BLACKBOX CCHFV IgG ELISA (15 serum pairs from CCHF patients, 12 sera from convalescent patients collected approximately one year after the infection), 15 a priori CCHFV-IgM/IgG negative control sera and 98 CCHFV IgM/IgG IIFT negative sera from healthy Kosovar blood donors were tested with both the VectoCrimean-CHF-IgM ELISA (**Fig 3C, Fig 4B, panel C in S1 Fig, S2 Fig, S3 Fig**) and the VectoCrimean-CHF-IgG ELISA (**Fig 3D, Fig 4B, panel D in S1 Fig, S2 Fig, S3 Fig**). While the BLACKBOX CCHFV IgM ELISA and the VectoCrimean-CHF-IgM ELISA gave identical results (**Table 6**), the BLACKBOX CCHFV IgG ELISA was found to be more efficient than the VectoCrimean-CHF-IgG ELISA (**Table 7**) in detecting IgG antibodies in the “late” serum samples collected between day 8 and day 36 (median: day 17) after onset of symptoms. To dissect this observation more thoroughly, the 30 paired “early” and “late” serum samples were classified according to the day after onset on which they were collected (**Fig 5**). While both the BLACKBOX CCHFV IgM ELISA (**Fig 5A, Table 8**) and the VectoCrimean-CHF-IgM ELISA (**Fig 5C, Table 8**) detected 100% of samples that were collected later than day 4 after onset of symptoms as positive (95% CI: 85.2%–100.0%), the BLACKBOX CCHFV IgG ELISA was significantly more sensitive than the VectoCrimean-CHF-IgG ELISA in detecting IgG antibodies in samples collected between day 11 and 19 after onset of symptoms (**Fig 5B, Fig 5D, Table 9;** BLACKBOX CCHFV IgG: 100% (95% CI: 71.5%–100.0%; VectoCrimean-CHF-IgG: 27% (95% CI: 6.0%–61.0%)).

**Table 8:** Analysis of CCHF patient samples, dependence from day after onset: Comparison of BLACKBOX CCHFV IgM ELISA, VectoCrimean-CHF IgM ELISA and in-house IgM IIFT results.

		IgM tested positive (# (%)) in			result statistical testing		
d.a.o.	# samples	IIFT	BB	VB	IIFT vs. BB	BB vs. VB	VB vs. IIFT
0–4	6	1 (17)	0 (0)	0 (0)	ns	ns	ns
≥ 5	24	24 (100)	24 (100)	24 (100)	ns	ns	ns

IIFT: indirect immune fluorescence test, BB: BLACKBOX CCHFV ELISA, VB: VectoCrimean-CHF ELISA. Statistical testing was performed using the Fisher’s exact test routine in GraphPad Prism. Ns: not significant (p > 0.05).

**Table 9:** Analysis of CCHF patient samples, dependence from day after onset: Comparison of BLACKBOX CCHFV IgG ELISA, VectoCrimean-CHF IgG ELISA and in-house IgG IIFT results.

		IgG tested positive (# (%)) in			result statistical testing		
d.a.o.	# samples	IIFT	BB	VB	IIFT vs. BB	BB vs. VB	VB vs. IIFT
0–4	6	1 (17)	0 (0)	0 (0)	ns	ns	ns
5–10	8	8 (100)	5 (62)	0 (0)	ns	*	***
11–19	11	11 (100)	11 (100)	3 (27)	ns	**	**
≥ 20	5	5 (100)	5 (100)	4 (80)	ns	ns	ns

IIFT: indirect immune fluorescence test, BB: BLACKBOX CCHFV ELISA, VB: VectoCrimean-CHF ELISA. Statistical testing was performed using the Fisher’s exact test routine in GraphPad Prism. Ns: not significant (p > 0.05); *: p < 0.05; **: p < 0.01; ***: p < 0.001.

### Correlation of IgM/IgG ELISA results with CCHFV IgM/IgG IIFT titers

**Fig 6:**
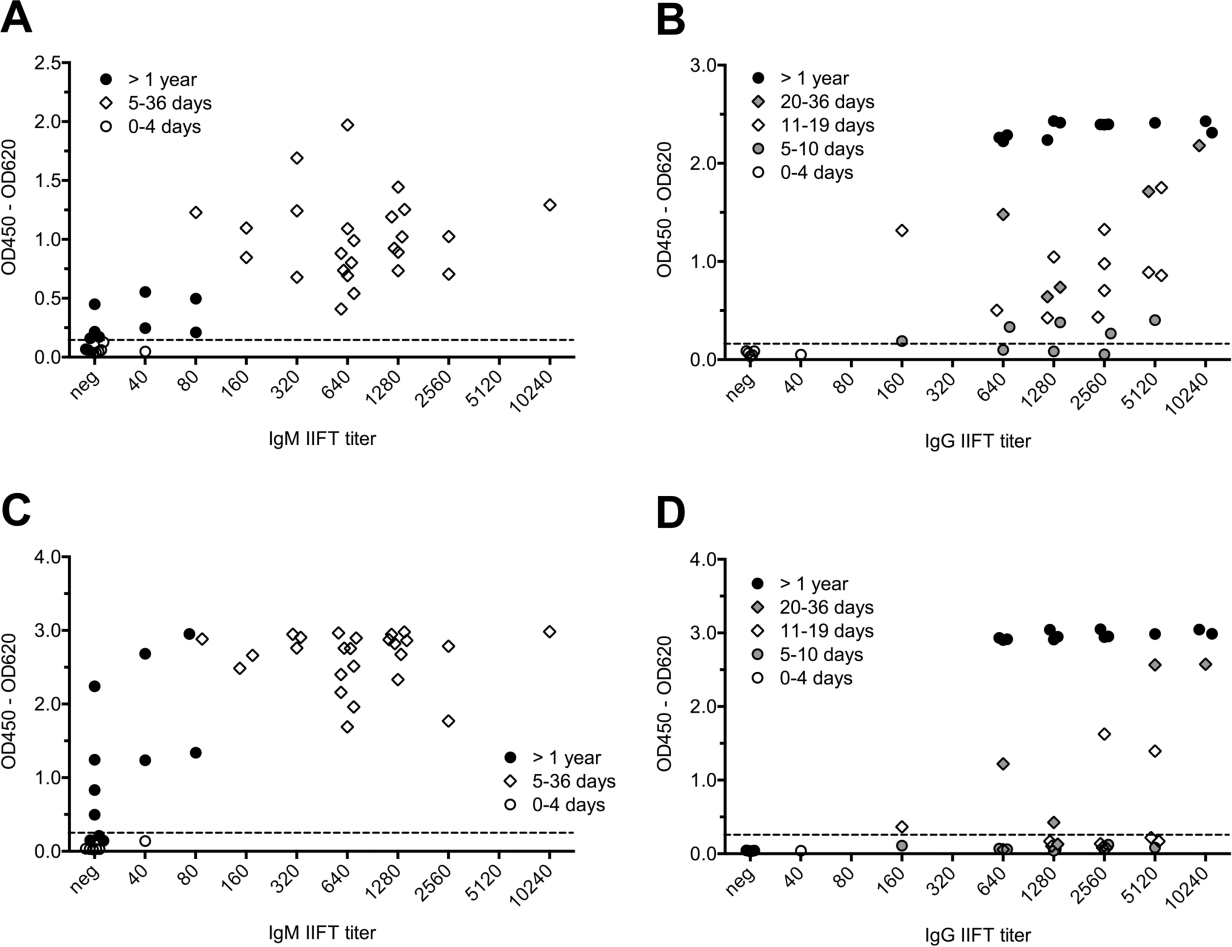
Analysis of CCHF patient samples: Raw data (OD450 – OD620), correlation with IIFT titer. **(A) BLACKBOX CCHFV IgM ELISA. (B) BLACKBOX CCHFV IgG ELISA. (C) VectoCrimean-CHF-IgM ELISA. (D) VectoCrimean-CHF-IgG ELISA.** 42 serum samples from 15 CCHF patients were classified according to their CCHFV IgM (A, C) and IgG IIFT titer (B, D), respectively. IIFT was performed using acetone-fixed Vero cells infected with CCHFV. Cut-off values (represented by dotted lines) were determined by ROC analysis ((A): 0.129, (B): 0.161) or according to the manufacturer’s instructions ((C): 0.240, (D): 0.246), respectively (see S2 Fig). Time of sampling (days after onset of symptoms) is indicated by the assigned symbols.

Evaluation of the correlation of optical densities measured using the BLACKBOX and Vector-Best IgM/IgG tests and the antibody titers obtained by in-house CCHFV IgM/IgG IIFT (**Fig 6**) revealed a marked difference between serum samples collected during the acute/early convalescent phase of the disease and follow-up samples collected approximately one year after the patients having overcome the disease. All follow-up samples generated a highly positive signal in the BLACKBOX CCHFV IgG test even a titer of 1:640, the lowest CCHFV IgG IIFT titer observed in these samples. On the other hand, optical densities measured for acute/early convalescent phase sera with the identical CCHFV IgG IIFT titer varied strongly from sample to sample (**Fig 6B**). Thereby, later sampling correlated with higher signals (**Fig 6B**).

## Discussion

In this work, two ELISA tests, a CCHFV μ-capture ELISA designated as “BLACKBOX CCHFV IgM ELISA” and a CCHFV IgG Immune Complex (IC) ELISA termed “BLACKBOX CCHFV IgG ELISA”, were developed employing the CCHFV ortholog of the major bunyavirus antigen, the nucleoprotein NP [12]. In the native virus particle, NP associates with the viral genomic RNA to form ribonucleoprotein (RNP) complexes, it is involved in virus assembly and mediates crucial interactions with host cell components like the cytoskeleton and the translation machinery [18]. Structural analysis of the 54 kDa CCHFV NP [18–20] revealed a racket-like structure with two distinct domains (“head” and “stalk”). Thereby, the stalk domain is formed by the central part of the 482 aa CCHFV NP polypeptide chain (≈ aa 180–300) and harbors the main antigenic region of the protein which is highly conserved between CCHFV strains from various endemic areas [21–23].

CCHFV NP has been used previously as an antigen in a variety of ELISA applications, either utilizing a combination of inactivated native virus lysate and an NP-specific monoclonal antibody [24] or purified recombinant NP produced either in insect cells [23,25,26], mammalian cells [27], *E*.*coli* [28,29] or even plants [30]. In these cases, most IgG ELISA applications were based on the concept of indirect ELISA [23,25–30], whereas IgM ELISA applications were set up using μ-capture protocols [24,27].

In contrast, the BLACKBOX CCHFV IgG ELISA developed in this work employs the IgG immune complex (IC) binding principle. In this type of ELISA, originally rheumatoid factor (RF) was used as a capture molecule to bind immune complexes formed between pathogen-specific IgG antibodies and either native or recombinant viral antigens [16,24,31–33]. Recently, we showed that the method could be further improved by replacing RF by the recombinantly produced tandem immunoglobulin-like domain of the human FcγR CD32 [17] and we have already demonstrated the potential of this by now patented assay format for the sensitive and specific detection of DENV-subtype-specific IgG antibodies [15].

In nature, CCHFV circulates in an enzootic tick-vertebrate-tick cycle and can be transmitted from livestock to human by contact with blood or other body fluids during activities like slaughtering [34]. Thus, ELISA tests for animal sera like the double-antigen sandwich ELISA recently developed by Sas *et al*. [35] are needed for surveillance purposes. The pronounced interspecies cross-reactivity of the recombinant CD32 fragment [17] may facilitate the development of such tests.

To evaluate the performance of the newly developed BLACKBOX CCHFV IgM/IgG ELISA tests, acute and convalescent phase sera from 15 patients with a PCR-confirmed CCHFV infection collected in Kosovo between 2013 and 2016 have been analyzed. Kosovo is a landlocked, southeastern European country on the central Balkan Peninsula with a population of 1.9 million. Due to a hot, dry climate favoring vector prevalence and extensive agricultural use, CCHFV is endemic in approximately half of its territory of 10,908 km^2^ [36]. Between 1954 and 2014, 304 confirmed cases of CCHF were reported with a case fatality rate (CFR) of 21% with the highest incidence in late spring/early summer (May–July) [37]. A seroprevalence of up to 9% was found in inhabitants of hyperendemic areas, the majority (70%) of seropositive individuals being males of advanced age (median age 62) who had probably been exposed to the virus during professional activities like farming or slaughtering [36]. Correspondingly, a high seroprevalence of up to 20% was observed in livestock (cows, goats, sheep) being kept in endemic areas [36]. Recently, analysis of tick species revealed *Hyalomma marginatum* as the dominant species (90% of all collected specimen) in endemic municipalities [38]. The percentage of infected *H*. *marginatum* ticks in endemic municipalities varied strongly between sampling sites (range 1.25%–29.4%, average 11%) [38]. Partial S segment sequences from all CCHFV-positive ticks could be classified as two different lineages, Kosovo I and II, in CCHFV clades VI (Europe 2) and V (Europe 1), respectively. Thereby, sequences isolated from *H*. *marginatum* ticks cluster with Kosovar human-derived sequences in clade V (Europe 1), suggesting that the Kosovo outbreaks are most likely due to *H*. *marginatum* transmitted CCHFV [38].

Results obtained with the BLACKBOX IgM/IgG ELISAs were compared with the respective in-house serological gold standard (CCHFV IgM/IgG IIFT) and commercial ELISA tests (VectoCrimean-CHF-IgM/IgG, Vector-Best, Russia) that have been extensively used for seroprevalence studies in various countries (e.g. Turkey [39–42], Greece [43–47], Bulgaria [48], Afghanistan [49], Kosovo [36], Tunisia [50], Ghana [51]) and for the diagnosis of acute cases [52,53]. Recently, a collaborative evaluation of both the VectoCrimean-CHF-IgG and the VectoCrimean-CHF-IgM test in five reference laboratories revealed a sensitivity of 87.8% (range 75.2%–95.3%) for the VectoCrimean-CHF-IgM test and a sensitivity of 80.4% (range 66.9%–90.2%) for the VectoCrimean-CHF-IgG test when compared with the respective in-house reference serological tests [3]. Specificities were found to be 98.9% (range 93.9%– 100.0%) for the VectoCrimean-CHF-IgM tests and 100% (range 95.8%–100%) for the VectoCrimean-CHF-IgG test [3]. Here, we found that both the newly developed BLACKBOX CCHFV IgM ELISA (employing recombinant CCHFV NP) and the VectoCrimean-CHF-IgM test readily detected CCHFV specific IgM antibodies in all samples collected after day 4 after onset of symptoms corresponding to a sensitivity of 100% when compared to the in-house gold standard CCHFV IgM IIFT. Both the newly developed BLACKBOX CCHFV IgG ELISA employing recombinant CCHFV NP and the VectoCrimean-CHF-IgG test generated strong positive results with serum samples originating from convalescent patients approximately one year after having overcome the disease. However, the BLACKBOX CCHFV IgG ELISA was found to show a superior sensitivity in the early detection of the rising IgG antibody titer in acutely ill patients: the BLACKBOX CCHFV IgG ELISA recognized, like the in-house gold standard IgG IIFT, all tested patient samples which were collected after day 11 after onset of symptoms as IgG positive, whereas only 43% of those samples tested positive in the VectoCrimean-CHF-IgG test. This increased sensitivity of the BLACKBOX CCHFV IgG ELISA may also be of importance for the main intended use of an IgG assay, that is seroprevalence studies.

When analyzing the correlation between CCHFV IgG IIFT titers and the OD values obtained using the BLACKBOX CCHFV IgG ELISA, a striking difference between serum samples collected during the acute/early convalescent phase of the disease and follow-up samples collected approximately one year after the patients having overcome the disease was observed. While the latter samples stably generated high OD values in the ELISA even at a rather low CCHFV IgG IIFT titer of 1:640, the signal heights of different samples with an identical CCHFV IgG IIFT titer varied strongly for acute/early convalescent phase samples. Thereby, higher signals correlated with later sampling. Thus, the very first IgG antibodies generated during acute CCHFV infection presumably do not predominantly recognize the CCHFV nucleoprotein but rather other antigens like the virus envelope glycoprotein G_c_ and therefore are not detectable with serological tests employing solely CCHFV NP as an antigen. A similar observation was made by Putkuri *et al*. [54] for a member of *orthobunyavirus* genus, the Inkoo virus (INKV). Here, the fraction of IgG antibodies recognizing either the nucleoprotein or the glycoprotein Gc, respectively, was found to differ considerably between serum samples from individuals with a long-standing immunity and samples collected during acute infection [54].

Both BLACKBOX CCHFV ELISAs employ the nucleoprotein of CCHFV strain Afg09-2990 (belonging to S segment lineage IV (Asia1), [55]) as antigen and were validated using CCHF serum samples originating from Kosovo. Thus, the patients’ infections were most likely caused by CCHFV strains belonging to S segment lineage V (Europe 1) [38,56]. To further assess the potential of the two BLACKBOX tests to detect anti-NP-antibodies induced by infections with CCHFV strains belonging to different S segment lineages, a sequence alignment of the nucleoprotein (NP) amino acid (aa) sequences of six CCHFV strains belonging to five different S segment lineages [55] was performed (**panels A and B in S4 Fig**). Sequence identities between the full length NP of the Afg09-2990 strain (lineage IV, Asia 1) and strains ArD157856 (lineage I, Africa 1), UG3010 (lineage II, Africa 2), ArD39554 (lineage III, Africa 3), Kosovo Hoti (lineage V, Europe 1) and Drosdov (lineage V (Europe 1)) were found to be 94.8%, 96.9%, 97.3%, 96.9% and 96.7%, respectively (**panel C in S4 Fig**). When the analysis was restricted to the NP stalk domain (aa 180–300) harboring the main antigenic region of the protein [21–23], even higher percentages of identity were observed with strains UG3010 (97.5%), ArD39554 (99.2%), Kosovo Hoti (99.2%), and Drosdov (99.2%) (**panel C in S4 Fig**). Therefore, the BLACKBOX tests most probably allow reliable detection of anti-CCHFV NP antibodies induced by CCHFV strains from different endemic regions of the world. This assumption is further supported by recent findings of Sas *et al*. [35] who demonstrated cross-detection of anti-CCHFV-NP antibodies in sera of animals infected with CCHFV strains belonging to S segment lineages III (Africa 3), IV (Asia 1), and V (Europe 1) in a double-antigen sandwich ELISA based on recombinant CCHFV NP derived from strain lbAr10300 (S segment lineage III (Africa 3)).

To our knowledge no information is publicly available on the precise nature of the antigen (native or recombinant antigen, CCHFV strain) used in the Vector-Best ELISAs. Nevertheless, Vanhomwegen *et al*. [3] cite a personal communication stating that the manufacturer validated the tests with a serum panel from CCHF cases originating from southwestern Russia. Therefore, we included a CCHFV strain originating from that region (Drosdov) in the alignment. Pairwise comparison of the full length NP sequence of the Kosovo Hoti strain with the Afg09-2990 NP (used as antigen in the BLACKBOX tests), the ArD39554 NP (full virus used in the IIFT tests) and the Drosdov NP revealed an identity of 96.9%, 96.9%, and 99.4%. When the analysis was restricted to the NP stalk domain, percentages were even higher (99.2%, 98.3%, and 100.0%). Thus, it is unlikely that the major reason for the observed differences in sensitivity of the BLACKBOX CCHFV IgG ELISA, the VectoCrimean-CHF-IgG ELISA and the CCHFV IgG IIFT is the use of CCHFV antigens resulting from different S segment lineages.

Due to the presence of all viral antigens in their native confirmation including potential posttranslational modifications, CCHFV IgG IIFT performed on CCHFV-infected Vero-cells is still the most sensitive method for detecting low levels of CCHFV-specific IgG antibodies in patient sera taken in the early phase of the disease. In addition, the obtained staining pattern allows the internal assessment of signal specificity. Nevertheless, application of this method requires a significant effort in the generation of assay components (mainly the infection of Vero cells with CCHFV under BSL-4 conditions), expensive and sensitive equipment (i.e. a fluorescence microscope) and last but not least specialized expertise for test evaluation. In contrast, ELISA components can be produced under BSL-1 conditions and, although assays have to be performed by trained lab personnel, no specialized expertise is necessary to evaluate the result.

Beside the competitive sensitivity and specificity of the BLACKBOX CCHFV ELISA tests, there are several advantages of the newly developed tests. First of all, the virus antigen can be produced in large amounts in *E*.*coli*; no cost-efficient eukaryotic expression system or cultivation of native virus is necessary. Furthermore, the BLACKBOX CCHFV IgG ELISA requires, due to the co-incubation of recombinant antigen and the patient serum, only one washing step and thus is easy to perform with only short hands-on times. Last but not least, all assay components are routinely produced and quality controlled in a specialized laboratory of the Bernhard Nocht Institute for Tropical Medicine (Hamburg, Germany) and thus can be easily made available to the scientific community.

In summary, two new serological tests (BLACKBOX CCHFV IgM and BLACKBOX CCHFV IgG) for the identification of acute CCHF cases and the performance of seroprevalence studies have been developed. The tests employ recombinant CCHFV NP as antigen and exhibit high reproducibility (Inter-/inter-assay and inter-laboratory variation) and competitive assay performance (sensitivity, specificity) in comparison with in-house gold standard testing by IIFT and commercially available test kits.

## Supporting information

S1 Checklist STARD checklist. (PDF)

S1 Fig Analysis of a priori CCHFV-IgM/IgG negative control sera and CCHFV IgM/IgG IIFT negative sera from healthy Kosovar blood donors. Serum samples were analyzed with the **BLACKBOX CCHFV IgM ELISA (A)**, the **BLACKBOX CCHFV IgG ELISA (B),** the **VectoCrimean-CHF-IgM ELISA (C)** and the **VectoCrimean-CHF-IgG ELISA (D).** Cut-off values (represented by dotted lines) were determined by ROC analysis ((A): 0.129, (B): 0.161) or according to the manufacturer’s instructions ((C): 0.240, (D): 0.246), respectively (see S2 Fig). Analyzed sera (A), (B): a priori CCHFV-IgM/IgG negative sera, n = 120: 49 HD Europe (EU), 14 HD Asia, 14 HD South America (SA), 10 Flavivirus infection (2 TBEV, 8 DENV), 33 malaria (27 *P*. *falciparum*, 6 *P*. *malariae*), 98 CCHFV IgM/IgG IIFT negative HD Kosovo (K); (C), (D): a priori CCHFV-IgM/IgG negative sera, n = 15: 3 HD EU, 4 HD Asia, 4 HD SA, 4 malaria (3 *P*. *falciparum*, 1 *P*. *malariae*), 98 CCHFV IgM/IgG IIFT negative HD K. HD: healthy blood donors.
(TIF)

S2 Fig ROC-analysis. A serum panel consisting of 30 paired serum samples from 15 CCHF patients, serum samples from 12 CCHF patients collected approximately one year after recovery from CCHFV infection and a set of a priori CCHFV IgM/IgG negative serum samples ((A), (B): n = 120; (C), (D): n = 15) was analyzed with the BLACKBOX CCHFV IgM ELISA, the BLACKBOX CCHFV IgG ELISA, the VectoCrimean-CHF-IgM ELISA and the VectoCrimean-CHF-IgG ELISA, see Fig 3 and S1 Fig. For the **BLACKBOX CCHFV IgM ELISA (A)**, ROC analysis was performed to determine the optimal cut-off (0.129, Youden index 1.000) for differentiation of PCR negative “early” (n = 5) and “late” samples (n = 14) from the negative samples (n = 120). For the **BLACKBOX CCHFV IgG ELISA (B)**, ROC analysis was performed to determine the optimal cut-off (0.161, Youden index 1.000) for differentiation of PCR negative “late” (n = 14) and “convalescent” samples (n = 12) from the negative samples (n = 120). ROC curves were generated accordingly for **the VectoCrimean-CHF-IgM ELISA (C)** and the **VectoCrimean-CHF-IgG ELISA (D)**. Displayed cut-offs were determined according to the manufacturer’s instruction ((C): 0.24, (D): 0.246)). Solid line: sensitivity (%), dotted line: specificity (%).
(TIF)

S3 Fig Analysis of paired CCHF patient samples: Seroconversion. A serum panel consisting of 30 paired serum samples from 15 CCHF patients and serum samples from 12 CCHF patients collected approximately one year after recovery from CCHFV infection was analyzed with the **BLACKBOX CCHFV IgM ELISA (A)**, the **BLACKBOX CCHF IgG ELISA (B)**, the **VectoCrimean-CHF-IgM ELISA (C)** and the **VectoCrimean-CHF-IgG ELISA (D)**. Solid lines connect results for samples originating from one and the same patient. Cut-off values (represented by dotted lines) were determined by ROC analysis ((A): 0.129, (B): 0.161) or according to the manufacturer’s instructions ((C): 0.240, (D): 0.246), respectively (see S2 Fig).
(TIF)

S4 Fig Lineage/strain variability of CCHFV nucleoprotein. **(A) GenBank accession numbers** of S segment nucleotide sequences and origin (country/host species) of CCHFV strains belonging to different S segment lineages. **(B) NP amino acid sequence alignment** for the CCHFV strains specified in (A). Bold: amino acid sequence of the CCHFV strain Afg09-2990 NP used as antigen in the BLACKBOX CCHFV IgM/IgG ELISA tests. Residues that are conserved between at least four of the six aligned strains are highlighted in gray. Conservative/non-conservative amino acid exchanges are marked in green/red. Blue background coloring indicates the NP stalk domain (aa 180–300). **(C) Pairwise NP aa sequence compariso**n for the CCHFV strains specified in (A). Numbers indicate the percentage of aa sequence identity (dark gray shading) and aa sequence similarity (light gray shading) of NP full length / stalk domain.
(TIF)

S1 Flowchart STARD flowchart. (PDF)
