# Supplementary material for: Sensitive and specific detection of Crimean-Congo Hemorrhagic Fever Virus (CCHFV)—Specific IgM and IgG antibodies in human sera using recombinant CCHFV nucleoprotein as antigen in μ-capture and IgG immune complex (IC) ELISA tests
Source: PLoS Negl Trop Dis. 2018 Mar 26;12(3):e0006366. doi: 10.1371/journal.pntd.0006366 (PMC5892944; doi:10.1371/journal.pntd.0006366)
Supplement: S1 Checklist — (PDF) [file pntd.0006366.s001.pdf]

Section & Topic	No	Item	Reported in section/paragraph
**TITLE OR ABSTRACT**			
	**1**	Identification as a study of diagnostic accuracy using at least one measure of accuracy (such as sensitivity, specificity, predictive values, or AUC)	Title<br>Abstract (paragraph 3)
**ABSTRACT**			
	**2**	Structured summary of study design, methods, results, and conclusions (for specific guidance, see STARD for Abstracts)	Abstract
**INTRODUCTION**			
	**3**	Scientific and clinical background, including the intended use and clinical role of the index test	Introduction
	**4**	Study objectives and hypotheses	Introduction (paragraph 3)
**METHODS**			
Study design	**5**	Whether data collection was planned before the index test and reference standard<br>were performed (prospective study) or after (retrospective study)	Materials and Methods (paragraph "Human sera")
Participants	**6**	Eligibility criteria	
	**7**	On what basis potentially eligible participants were identified (such as symptoms, results from previous tests, inclusion in registry)	
	**8**	Where and when potentially eligible participants were identified (setting, location and dates)	
	**9**	Whether participants formed a consecutive, random or convenience series	
Test methods	**10a**	Index test, in sufficient detail to allow replication	Materials and Methods (paragraphs "IgM µ-capture ELISA (BLACKBOX CCHFV IgM)" and "IgG Immune-Complex (IC) ELISA (BLACKBOX CCHFV IgG)"
	**10b**	Reference standard, in sufficient detail to allow replication	Materials and Methods (paragraphs "VectoCrimean-CHF-IgM/IgG ELISA …" and "Human sera/CCHF patient sera")
	**11**	Rationale for choosing the reference standard (if alternatives exist)	na
	**12a**	Definition of and rationale for test positivity cut-offs or result categories of the index test, distinguishing pre-specified from exploratory	Results (paragraph "Determination of optimal assay cut-offs …")
	**12b**	Definition of and rationale for test positivity cut-offs or result categories of the reference standard, distinguishing pre-specified from exploratory	Materials and Methods (paragraph "VectoCrimean-CHF-IgM/IgG ELISA …")
	**13a**	Whether clinical information and reference standard results were available to the performers/readers of the index test	Materials and Methods (paragraph "Human sera/CCHF patient sera")
	**13b**	Whether clinical information and index test results were available to the assessors of the reference standard	
Analysis	**14**	Methods for estimating or comparing measures of diagnostic accuracy	Materials and Methods (paragraph "Data analysis")
	**15**	How indeterminate index test or reference standard results were handled	Materials and Methods (paragraph "VectoCrimean-CHF-IgM/IgG ELISA …")
	**16**	How missing data on the index test and reference standard were handled	na
	**17**	Any analyses of variability in diagnostic accuracy, distinguishing pre-specified from exploratory	na
	**18**	Intended sample size and how it was determined	na
**RESULTS**			
Participants	**19**	Flow of participants, using a diagram	See Flowchart S1
	**20**	Baseline demographic and clinical characteristics of participants	Materials and Methods (paragraph "Human sera/CCHF patient sera")
	**21a**	Distribution of severity of disease in those with the target condition	na
	**21b**	Distribution of alternative diagnoses in those without the target condition	na
	**22**	Time interval and any clinical interventions between index test and reference standard	na
Test results	**23**	Cross tabulation of the index test results (or their distribution) by the results of the reference standard	See Figs. 3 and 5

	**24**	Estimates of diagnostic accuracy and their precision (such as 95% confidence intervals)	Abstract, Results (paragraphs "Determination of optimal assay cut-offs …") and "Comparison to commercially available ELISA tests …")
	**25**	Any adverse events from performing the index test or the reference standard	na
**DISCUSSION**			
	**26**	Study limitations, including sources of potential bias, statistical uncertainty, and generalisability	Introduction (paragraph 1), Discussion (paragraph 1)
	**27**	Implications for practice, including the intended use and clinical role of the index test	Discussion (paragraph 7)
**OTHER INFORMATION**			
	**28**	Registration number and name of registry	na
	**29**	Where the full study protocol can be accessed	na
	**30**	Sources of funding and other support; role of funders	See Financial Disclosure section of submission form
