# Supplementary figures and images for: Sensitive and specific detection of Crimean-Congo Hemorrhagic Fever Virus (CCHFV)—Specific IgM and IgG antibodies in human sera using recombinant CCHFV nucleoprotein as antigen in μ-capture and IgG immune complex (IC) ELISA tests
Source: PLoS Negl Trop Dis. 2018 Mar 26;12(3):e0006366. doi: 10.1371/journal.pntd.0006366 (PMC5892944; doi:10.1371/journal.pntd.0006366)

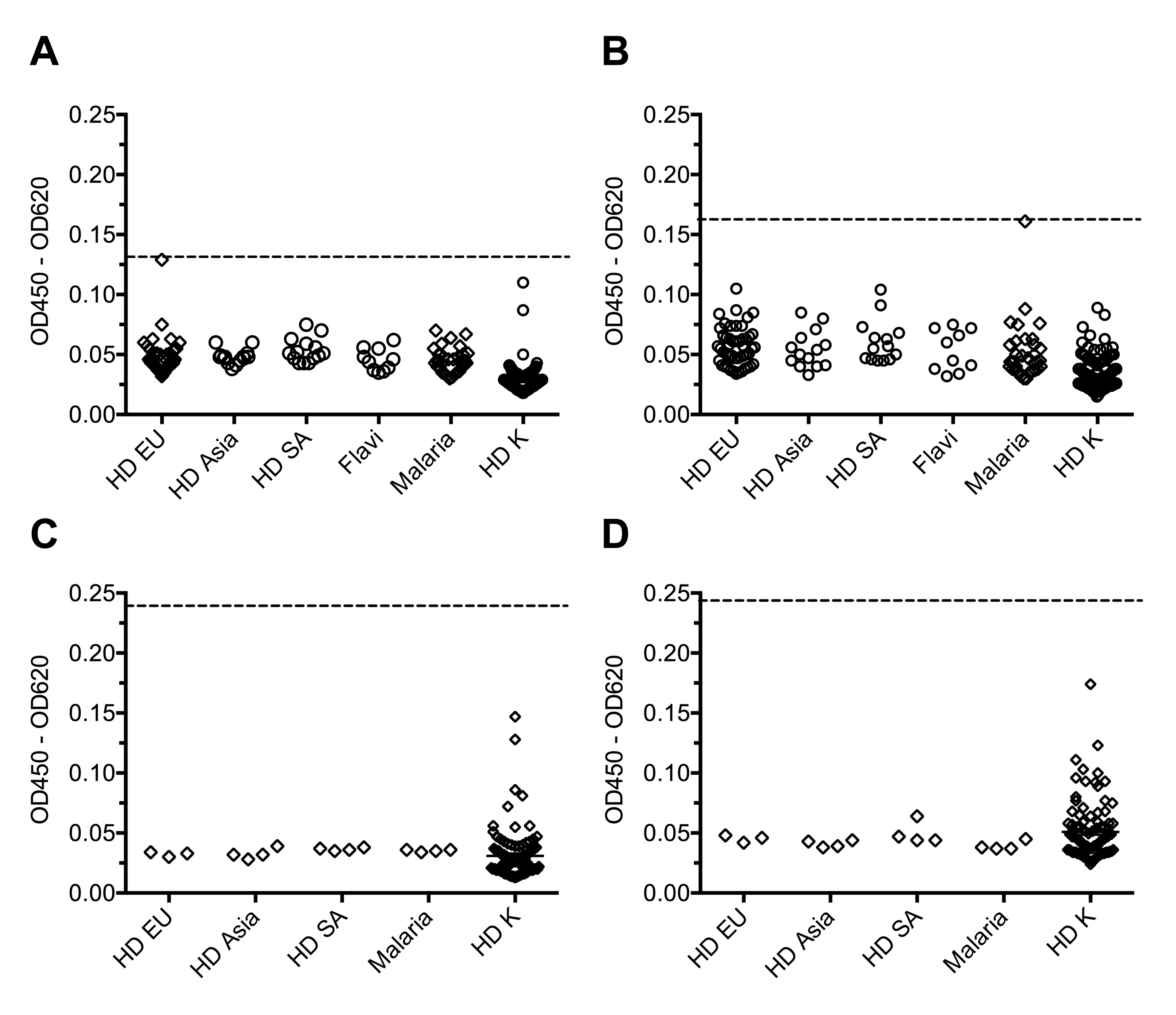

Supplement: S1 Fig — Serum samples were analyzed with the BLACKBOX CCHFV IgM ELISA (A), the BLACKBOX CCHFV IgG ELISA (B), the VectoCrimean-CHF-IgM ELISA (C) and the VectoCrimean-CHF-IgG ELISA (D). Cut-off values (represented by dotted lines) were determined by ROC analysis ((A): 0.129, (B): 0.161) or according to the manufacturer’s instructions ((C): 0.240, (D): 0.246), respectively (see S2 Fig). Analyzed sera (A), (B): a priori CCHFV-IgM/IgG negative sera, n = 120: 49 HD Europe (EU), 14 HD Asia, 14 HD South America (SA), 10 Flavivirus infection (2 TBEV, 8 DENV), 33 malaria (27 P. falciparum, 6 P. malariae), 98 CCHFV IgM/IgG IIFT negative HD Kosovo (K); (C), (D): a priori CCHFV-IgM/IgG negative sera, n = 15: 3 HD EU, 4 HD Asia, 4 HD SA, 4 malaria (3 P. falciparum, 1 P. malariae), 98 CCHFV IgM/IgG IIFT negative HD K. HD: healthy blood donors. (TIF) [file pntd.0006366.s002.tif]

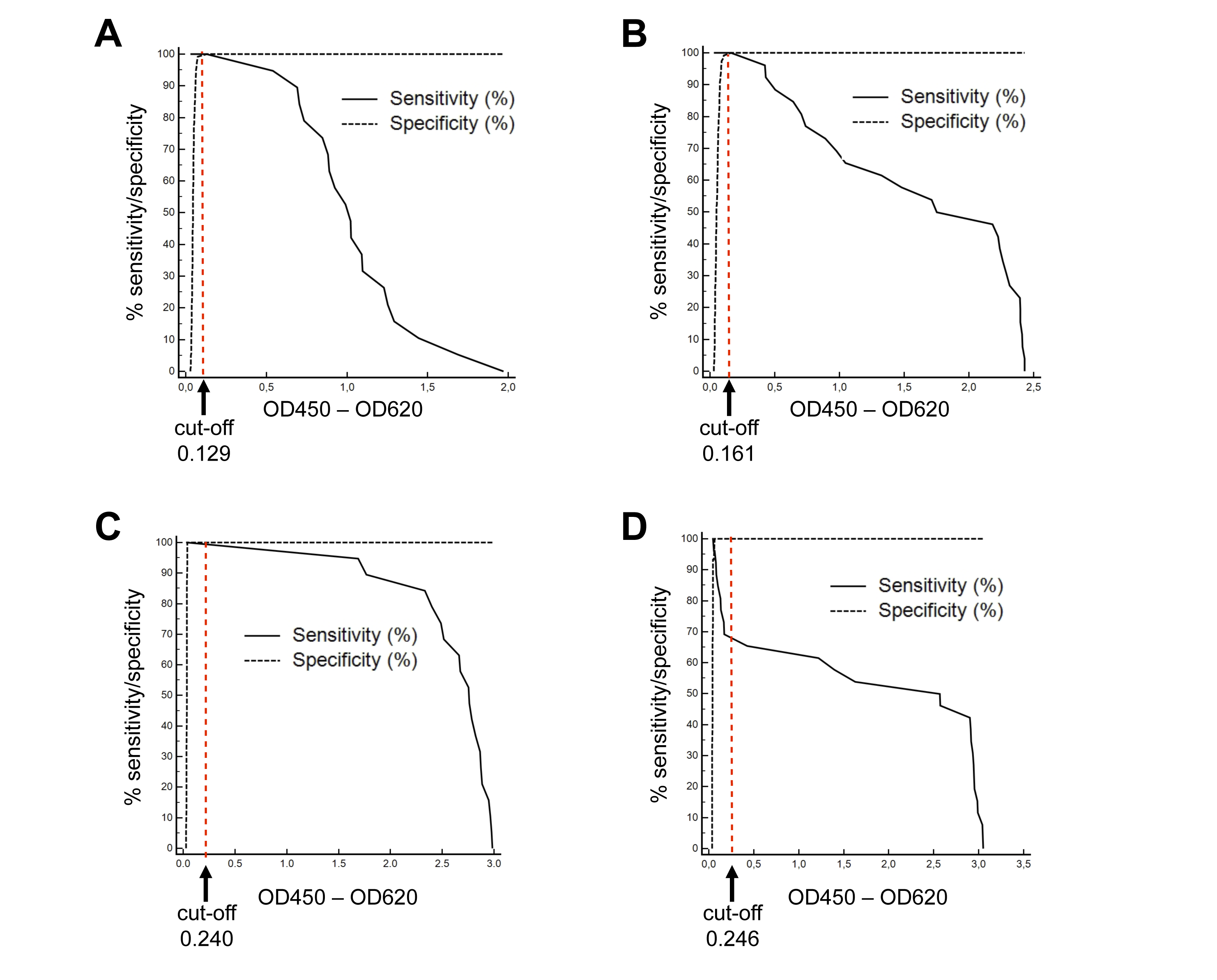

Supplement: S2 Fig — A serum panel consisting of 30 paired serum samples from 15 CCHF patients, serum samples from 12 CCHF patients collected approximately one year after recovery from CCHFV infection and a set of a priori CCHFV IgM/IgG negative serum samples ((A), (B): n = 120; (C), (D): n = 15) was analyzed with the BLACKBOX CCHFV IgM ELISA, the BLACKBOX CCHFV IgG ELISA, the VectoCrimean-CHF-IgM ELISA and the VectoCrimean-CHF-IgG ELISA, see Fig 3 and S1 Fig. For the BLACKBOX CCHFV IgM ELISA (A), ROC analysis was performed to determine the optimal cut-off (0.129, Youden index 1.000) for differentiation of PCR negative “early” (n = 5) and “late” samples (n = 14) from the negative samples (n = 120). For the BLACKBOX CCHFV IgG ELISA (B), ROC analysis was performed to determine the optimal cut-off (0.161, Youden index 1.000) for differentiation of PCR negative “late” (n = 14) and “convalescent” samples (n = 12) from the negative samples (n = 120). ROC curves were generated accordingly for the VectoCrimean-CHF-IgM ELISA (C) and the VectoCrimean-CHF-IgG ELISA (D). Displayed cut-offs were determined according to the manufacturer’s instruction ((C): 0.24, (D): 0.246)). Solid line: sensitivity (%), dotted line: specificity (%). (TIF) [file pntd.0006366.s003.tif]

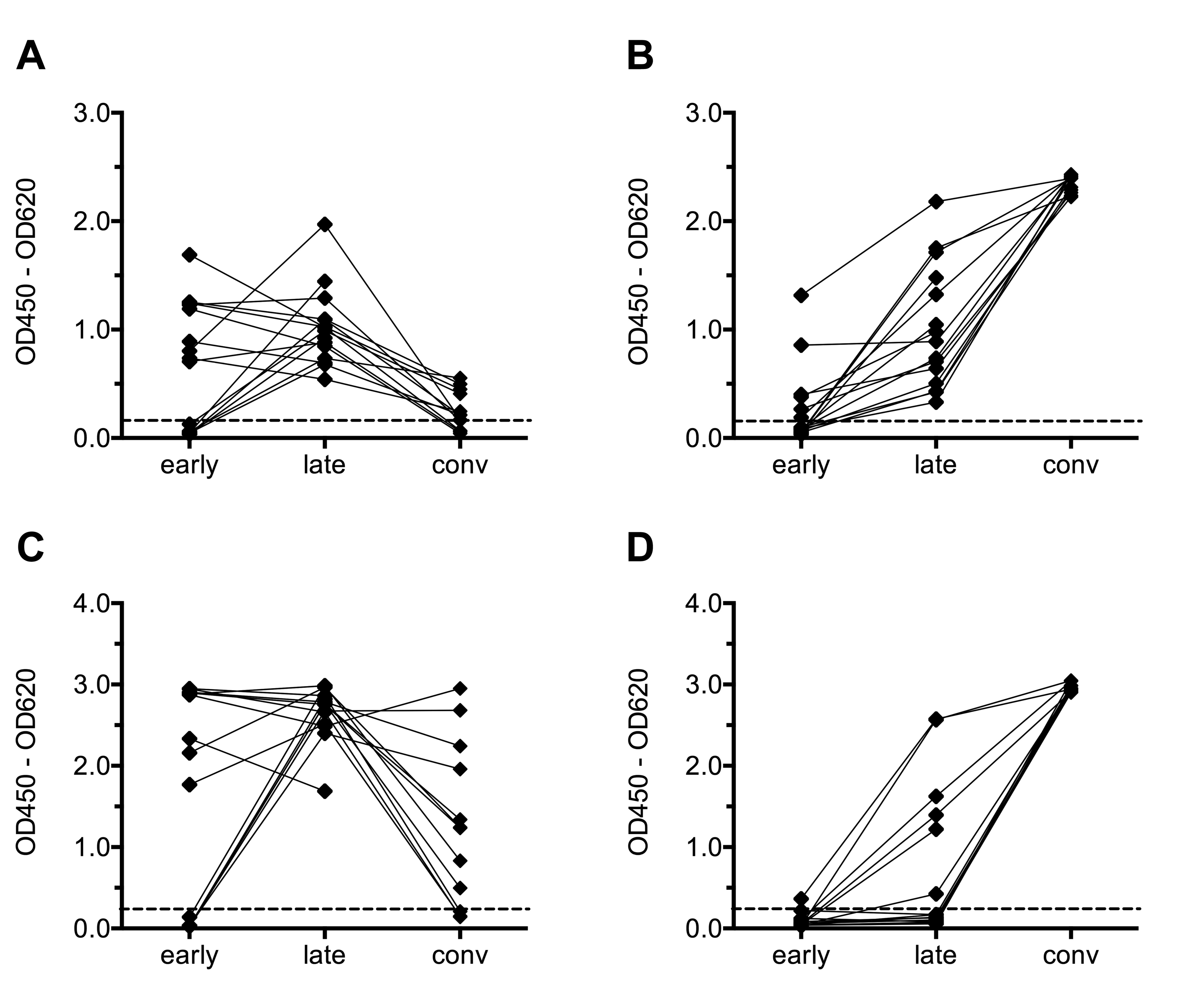

Supplement: S3 Fig — A serum panel consisting of 30 paired serum samples from 15 CCHF patients and serum samples from 12 CCHF patients collected approximately one year after recovery from CCHFV infection was analyzed with the BLACKBOX CCHFV IgM ELISA (A), the BLACKBOX CCHF IgG ELISA (B), the VectoCrimean-CHF-IgM ELISA (C) and the VectoCrimean-CHF-IgG ELISA (D). Solid lines connect results for samples originating from one and the same patient. Cut-off values (represented by dotted lines) were determined by ROC analysis ((A): 0.129, (B): 0.161) or according to the manufacturer’s instructions ((C): 0.240, (D): 0.246), respectively (see S2 Fig). (TIF) [file pntd.0006366.s004.tif]

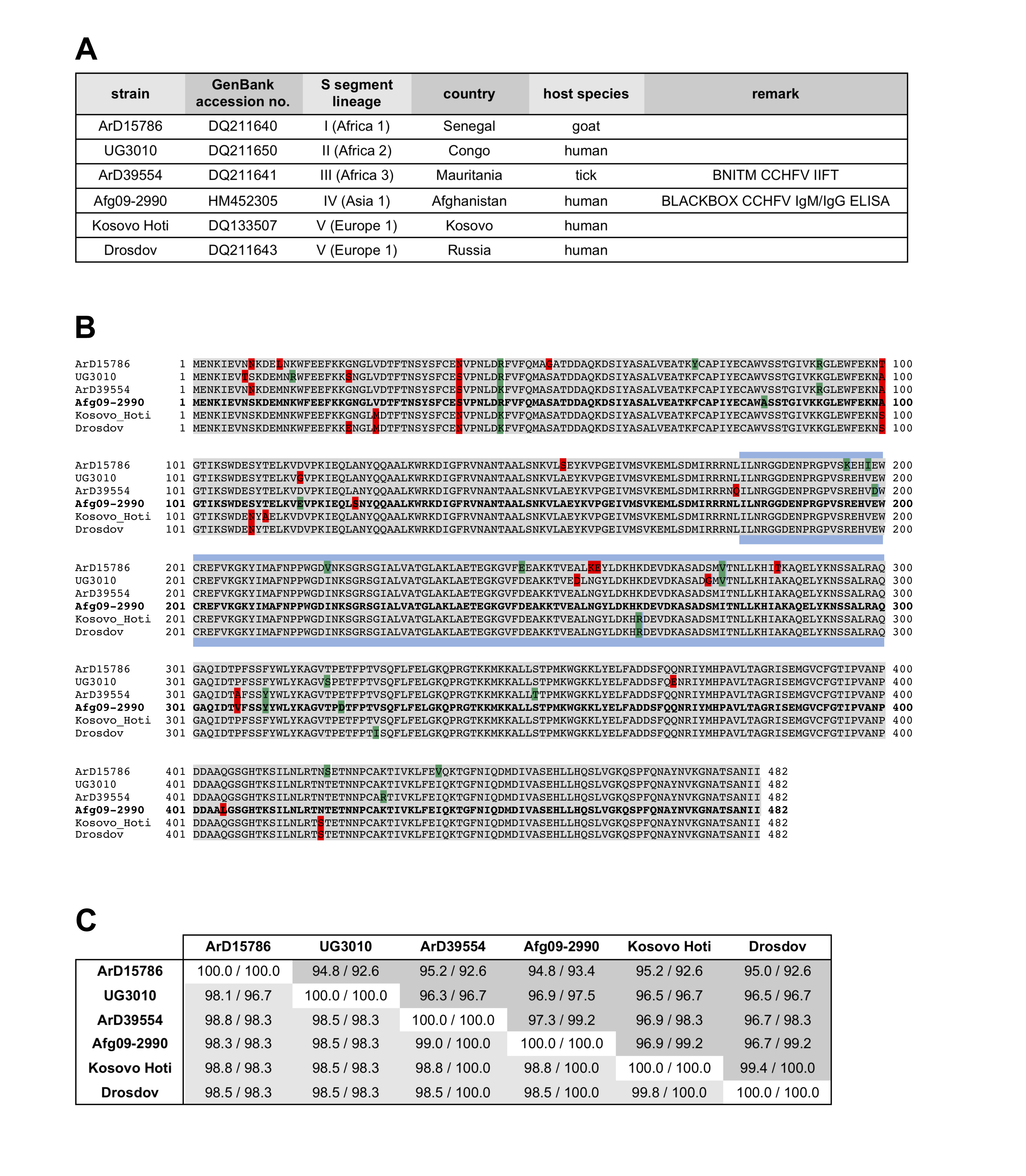

Supplement: S4 Fig — (A) GenBank accession numbers of S segment nucleotide sequences and origin (country/host species) of CCHFV strains belonging to different S segment lineages. (B) NP amino acid sequence alignment for the CCHFV strains specified in (A). Bold: amino acid sequence of the CCHFV strain Afg09-2990 NP used as antigen in the BLACKBOX CCHFV IgM/IgG ELISA tests. Residues that are conserved between at least four of the six aligned strains are highlighted in gray. Conservative/non-conservative amino acid exchanges are marked in green/red. Blue background coloring indicates the NP stalk domain (aa 180–300). (C) Pairwise NP aa sequence comparison for the CCHFV strains specified in (A). Numbers indicate the percentage of aa sequence identity (dark gray shading) and aa sequence similarity (light gray shading) of NP full length / stalk domain. (TIF) [file pntd.0006366.s005.tif]

## Flowchart S1: Flow of participants

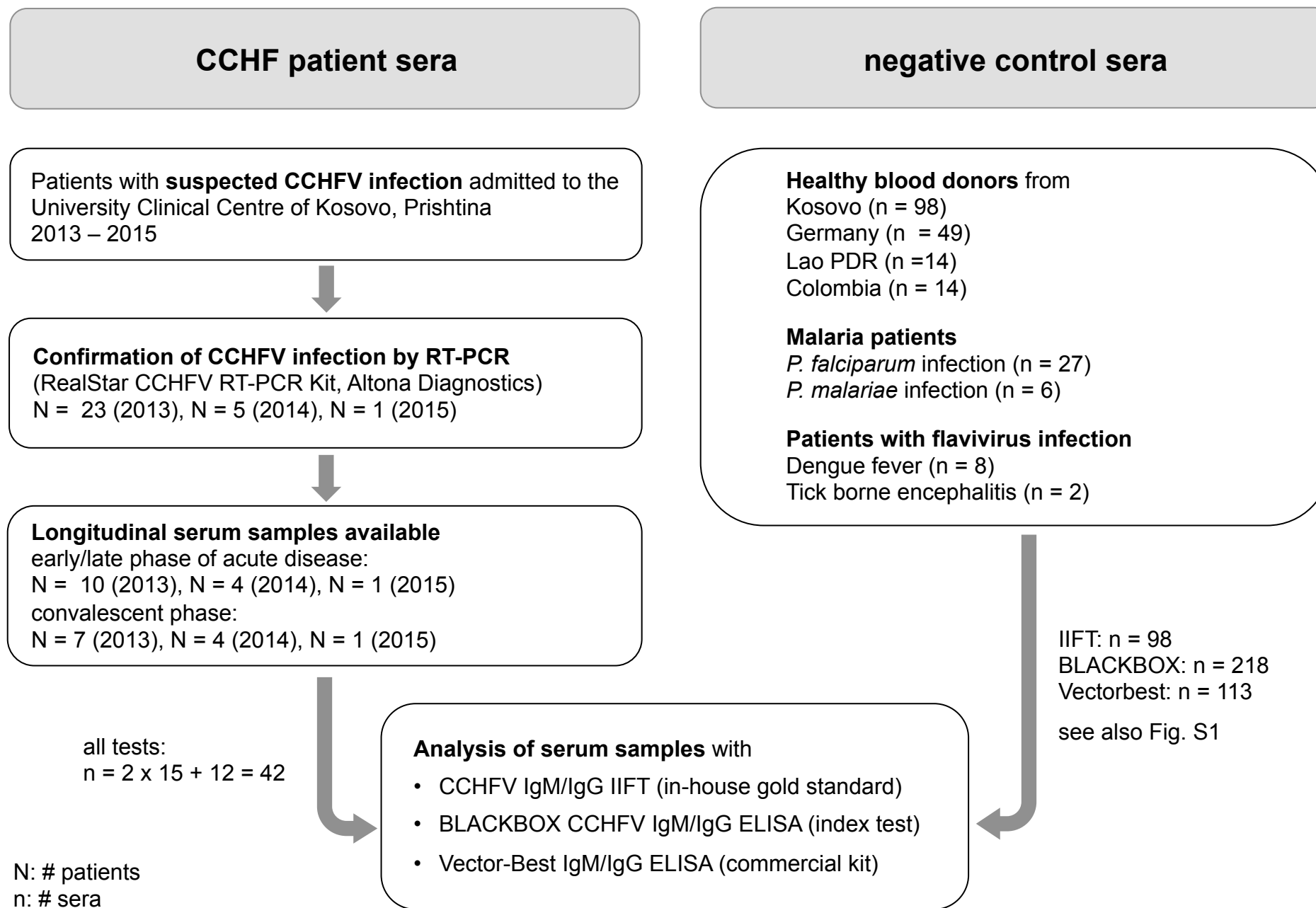

Supplement: S1 Flowchart — (PDF) [file pntd.0006366.s006.pdf]
